# Genomic Resources to Guide Improvement of the Shea Tree

**DOI:** 10.3389/fpls.2021.720670

**Published:** 2021-09-09

**Authors:** Iago Hale, Xiao Ma, Arthur T. O. Melo, Francis Kwame Padi, Prasad S. Hendre, Sarah B. Kingan, Shawn T. Sullivan, Shiyu Chen, Jean-Marc Boffa, Alice Muchugi, Agyemang Danquah, Michael Teye Barnor, Ramni Jamnadass, Yves Van de Peer, Allen Van Deynze

**Affiliations:** ^1^Department of Agriculture, Nutrition, and Food Systems, University of New Hampshire, Durham, NH, United States; ^2^Department of Plant Biotechnology and Bioinformatics, Ghent University, Ghent, Belgium; ^3^Center for Plant Systems Biology, VIB, Ghent, Belgium; ^4^Plant Breeding Division, Cocoa Research Institute of Ghana, Ghana Cocoa Board, New Tafo, Ghana; ^5^AOCC Genomics Laboratory and Tree Genebank Research Unit, World Agroforestry (CIFOR-ICRAF), Nairobi, Kenya; ^6^Pacific Biosciences, Menlo Park, CA, United States; ^7^Phase Genomics, Seattle, WA, United States; ^8^Seed Biotechnology Center, University of California, Davis, Davis, CA, United States; ^9^The Forage Genebank, Feed and Forage Development Program, International Livestock Research Institute, Addis Ababa, Ethiopia; ^10^West Africa Centre for Crop Improvement, College of Basic and Applied Sciences, University of Ghana, Accra, Ghana; ^11^College of Horticulture, Academy for Advanced Interdisciplinary Studies, Nanjing Agricultural University, Nanjing, China; ^12^Centre for Microbial Ecology and Genomics, Department of Biochemistry, Genetics and Microbiology, University of Pretoria, Pretoria, South Africa

**Keywords:** shea tree, *Vitellaria paradoxa*, reference genome, fatty acids, SNPs, whole genome duplication, plant breeding

## Abstract

A defining component of agroforestry parklands across Sahelo-Sudanian Africa (SSA), the shea tree (*Vitellaria paradoxa*) is central to sustaining local livelihoods and the farming environments of rural communities. Despite its economic and cultural value, however, not to mention the ecological roles it plays as a dominant parkland species, shea remains semi-domesticated with virtually no history of systematic genetic improvement. In truth, shea’s extended juvenile period makes traditional breeding approaches untenable; but the opportunity for genome-assisted breeding is immense, provided the foundational resources are available. Here we report the development and public release of such resources. Using the FALCON-Phase workflow, 162.6 Gb of long-read PacBio sequence data were assembled into a 658.7 Mbp, chromosome-scale reference genome annotated with 38,505 coding genes. Whole genome duplication (WGD) analysis based on this gene space revealed clear signatures of two ancient WGD events in shea’s evolutionary past, one prior to the Astrid-Rosid divergence (116–126 Mya) and the other at the root of the order Ericales (65–90 Mya). In a first genome-wide look at the suite of fatty acid (FA) biosynthesis genes that likely govern stearin content, the primary determinant of shea butter quality, relatively high copy numbers of six key enzymes were found (*KASI*, *KASIII*, *FATB*, *FAD2*, *FAD3*, and *FAX2*), some likely originating in shea’s more recent WGD event. To help translate these findings into practical tools for characterization, selection, and genome-wide association studies (GWAS), resequencing data from a shea diversity panel was used to develop a database of more than 3.5 million functionally annotated, physically anchored SNPs. Two smaller, more curated sets of suggested SNPs, one for GWAS (104,211 SNPs) and the other targeting FA biosynthesis genes (90 SNPs), are also presented. With these resources, the hope is to support national programs across the shea belt in the strategic, genome-enabled conservation and long-term improvement of the shea tree for SSA.

## Introduction

Shea tree (*Vitellaria paradoxa*) is a unique agroforestry tree species central to sustaining local livelihoods and the farming environments of rural communities across Africa’s Sudano-Sahelian agroclimactic belt. Cited as the second most important oil crop in Africa after oil palm, shea tree is likely among the most economically and culturally important indigenous tree species in the Sudano-Sahelian region of Africa where oil palm does not grow (Hall and Tomlinson, 1996). Rural families in hundreds of thousands of villages across the so-called “shea belt,” a 500–750 km wide semi-arid area stretching 6,000 km and spanning 21 countries from Senegal to South Sudan, use shea in their daily lives as an edible butter/oil, soap, cosmetic, and medicine. Shea is also a multimillion dollar export commodity as an ingredient in luxury cosmetic and personal care (e.g., moisturizing creams, sun lotions, and soaps) and pharmaceutical (e.g., cholesterol-lowering and anti-arthritic remedies) products. The largest export demand (∼90%) for shea is, however, linked to the extraction of edible stearin used in the formulation of cocoa butter equivalents (CBE) for chocolate confectionary (Rousseau et al., 2015).

As an indigenous tree species, *V. paradoxa* is traditionally not planted because of its extensive juvenile period (commonly 10–25 years) and local abundance, not to mention the tenurial challenges associated with being unable to differentiate naturally regenerated from planted trees. Instead, shea agroforestry parklands, comprised of annual crops and scattered shea trees that can reach densities of 20–50 trees/ha in areas of strong shea culture, result from self-sown propagation and systematic management (selection and protection, as opposed to planting) by farmers through successive fallow and cultivation cycles. Farmer selection of preferred individuals and removal of inferior trees for charcoal production or building materials serve to increase the levels of locally valued traits, resulting in what has been called a semi-domestication of the species (Lovett and Haq, 2000; Maranz and Wiesman, 2003).

Based on population and daily shea butter consumption data, the number of women taking part in shea nut collection across the shea belt is estimated at more than 18 million (Naughton et al., 2015). Indeed, shea has been called “women’s gold” because it is one of only a few resources that female members of rural households in the region have control over, from harvesting to commercialization (Elias and Carney, 2007). The sale of shea products allows women to secure additional food for themselves and their children once cereal harvests are exhausted (Pouliot, 2012) and to generate cash for household expenses including clothing, medicine, and school fees. The seasonality of shea availability is also critical to farming households’ nutrition. Shea fruits are the only widely available, energy-rich food source at the time when land is tilled and crops are planted at the end of the dry season. The fruits supply significant amounts of protein, sugar, calcium, potassium, and essential fatty acids (Honfo et al., 2014) during this annual ‘hungry season’ when cereal stocks in granaries are lowest and labor requirements for field preparations with the coming of the rains are highest (Maranz et al., 2004a).

Using a land suitability model parameterized with conservative tree density estimates, Naughton et al. (2015) estimated that there are 1.84 billion shea trees over its distribution range, making it one of the largest populations of an economic tree species in the region, not to mention a source of considerable environmental benefits. In high production areas, such as those found in northern Ghana and Burkina Faso’s Central Plateau, shea can account for up to 80% of tree populations and a large portion of the standing biomass of the farmed parklands (Boffa, 1999). These parkland systems, edified through human management, contain substantial carbon stores with enormous potential to mitigate climate change via future carbon sequestration (Takimoto et al., 2009; Luedeling and Neufeldt, 2012). Currently, the shea value chain is estimated to fix about 1.5 million tons of CO_2_ equivalent (tCO_2_-e) yearly, or a negative carbon footprint of 1.04 tCO_2_-e per ton of shea kernels produced (Bockel et al., 2020).

Despite shea’s widespread importance to rural livelihoods (especially those of women), its growing export market, and its ecological role as a key species in these agroforestry parkland systems, the sustainability of *V. paradoxa* populations is threatened by increasing demographic pressure and the quickening spread of mechanized farming practices. This raises concerns for possible future production gaps. Given increasing saturation of arable land, cultivation periods are extended and fallow intervals, which were traditionally responsible for the recruitment of young shea trees (Ræbild et al., 2012), are reduced or altogether abandoned. As a result, shea tree densities have been declining for decades; trees have been aging and regeneration is low (Gijsbers et al., 1994; Boffa, 1999). Farmers collecting shea nuts need to travel longer distances, increasing the labor required for nut collection; and when monetary returns to collectors drop, farmers begin to convert shea parklands to plantations of alternative tree crops, such as cashew and mango. Also, the relatively recent emphasis on large-scale land investment and agricultural development projects aiming to intensify maize, cotton, and biofuel production with mechanized farming are an additional threat to the retention of shea trees in farmers’ fields (Poudyal, 2011). Elsewhere in areas of high energy demand, competing uses of shea in parklands or uncultivated woodlands can be favored, such as tree cutting for firewood and charcoal making, leading to the degradation of shea stands.

Notwithstanding these pressures, shea consistently features at the top of farmers’ lists of priority species; and surveys indicate that farmers would plant shea trees if improved varieties were available and visually distinguishable (for purposes of clarifying ownership) from naturally regenerated trees (Poudyal, 2011). Like all high-value fruit trees planted in these systems, deliberately planted shea stands could be established in closer proximity to household compounds, thus reducing walking and collection times. Furthermore, the introduction of improved shea varieties has the potential to ease harvesting, boost and reduce tree-to-tree variation in yield and quality, and improve land and labor use efficiency, especially if combined with improved cultural practices. Various recent efforts to distribute shea seedlings top-worked with scions of identified “plus” (i.e., superior) *in situ* accessions signify a felt need to improve upon the passive semi-domestication process, even if it requires a fundamental shift in the traditional planting culture.

Stearin buyers on the international market also clamor for improved genetics. With stearic and oleic acids constituting nearly 90% of kernel fat content (Maranz et al., 2004b; Ugese et al., 2010), shea is one of the few economically viable natural sources of stearic–oleic–stearic triacylglycerols, a valued vegetable stearin due to its comparable functional properties to cocoa butter (Allal et al., 2013). But while it is the preferred stearin source for CBE production, the quality, quantity and price of the gathered shea crop are criticized by stearin buyers as erratic (LMC International, 2014). This comes as no surprise, as the out-crossing reproductive physiology of the species results in naturally regenerating heterozygous populations with highly variable biochemical profiles. A genetic improvement program for shea is therefore viewed as a promising means of achieving higher resource supply consistency and quality, with the positive effect of reducing price fluctuations and satisfying industry demands.

In short, the systematic genetic improvement of the shea tree is strongly justified to address both the threats to and rising demand for the resource, as well as development opportunities in the sector; yet there has been virtually no history of such work to date. The body of research relevant to the task, however, is extensive and indicates great promise for progress. In the mid-20th century, pioneering cytogenetic and oil/butter compositional studies on *V. paradoxa* (then *Butyrospermum parkii*) were conducted, describing the species as a diploid (2n = 24) (Miège, 1954) and providing the first insights into the fatty acid composition of shea butter (Hilditch and Saletore, 1931), already well-recognized for its cultural importance and economic potential (Chevalier, 1948). But it is within the last 30 years that a surge in shea research has occurred, particularly in the form of distribution, parkland regeneration, and diversity studies. Lovett and Haq (2000) published the results of an isozyme-based diversity analysis of shea in Ghana, signaling a shift from methods based on morphological traits to those based on molecular markers. A suite of similar studies followed, some of impressive pan-African scope (e.g., Bouvet et al., 2004; Allal et al., 2011), making use of various low-density sets of markers, including SSRs (8–14), RAPDs (67), chloroplastic SNPs (4), or combinations thereof (see e.g., Fontaine et al., 2004; Kelly et al., 2004; Cardi et al., 2005; Sanou et al., 2005; Allal et al., 2008; Logossa et al., 2011; Gwali et al., 2015; Abdulai et al., 2017). The conclusions of such studies were consistent. Shea is a weakly selfing (low fixation index) species characterized by moderate to high heterozygosity and extensive gene flow. With the Dahomey Gap serving as a dividing boundary, there is strong genetic differentiation between stearic-rich subspecies *paradoxa* in the West and oleic-rich *nilotica* in the East, with relatively higher allelic richness observed in western populations; yet essentially no phylogeographic signal is observed within regions. Such weak intraregional differentiation, manifested by the majority (85–95%) of genetic variation existing within populations, is expected in outcrossing, long-lived trees of widespread occurrence over a continuous range and further suggests little impact of domestication on genetic diversity.

In addition to their shared insights, there is a notable limitation common among these studies as well, namely the inability to associate low-resolution genetic data with factors relevant to shea improvement and cultivation, whether it be ethnovarietal attributes, fatty acid profiles, or basic growth parameters. To this end, such genetic investigations echo the patterns of diversity established by the numerous phenotypic studies of the species. Starting with Chevalier’s first observations in the 1940’s, shea diversity studies based on morphological and chemical profile data consistently reported high trait variability, with most (60–90%) of that variation existing within populations (see e.g., Sanou et al., 2005; Akihisa et al., 2011; Allal et al., 2013). Productivity exhibits a similarly high variation within populations. Desmarest (1958), for example, observed of a natural population in Burkina Faso that over half of the trees had essentially no economic value. The work of Boffa et al. (1996) supports that basic conclusion, noting that 24% of the trees they observed accounted for 55% of total stand production. In sum, phenotypic studies to date indicate the existence of substantial genetic variation in economically relevant traits within highly diverse populations, the same populations which defy dissection via low-density molecular markers. In comparing methods for estimating heritability of various foliar traits in shea, Bouvet et al. (2008) observed that a method relying on marker-based estimates of relatedness (12 SSRs) failed to detect the significant heritabilities revealed by a traditional pedigree-based method. Their conclusion was that new approaches are needed for marker-based methods in an agroforestry species like shea. Fortunately, in the intervening years, tremendous strides have been made in the development of such methods. Indeed, genome-wide association studies (GWAS) in unstructured populations have become common in many species (Cortes et al., 2021), provided the supporting genomic resources, including a well-annotated reference genome and sufficiently dense marker sets, are available.

Conversion through genetic improvement of this semi-domesticated agroforestry species into an early fruiting, highly productive tree displaying desired characteristics (e.g., consistent fruit and nut quality, tree architecture, yield stability, etc.) is required to trigger the shift to a more deliberate and active conservation and deployment of shea genetic diversity. Shea’s extended juvenile period makes traditional breeding approaches untenable for this task, but the opportunity for genome-assisted improvement is immense. The resources developed in this study are intended to support such a new era of research, one requiring high genomic resolution commensurate with shea’s well established patterns of diversity.

## Materials and Methods

### Plant Genetic Resources

**FIGURE 1:**
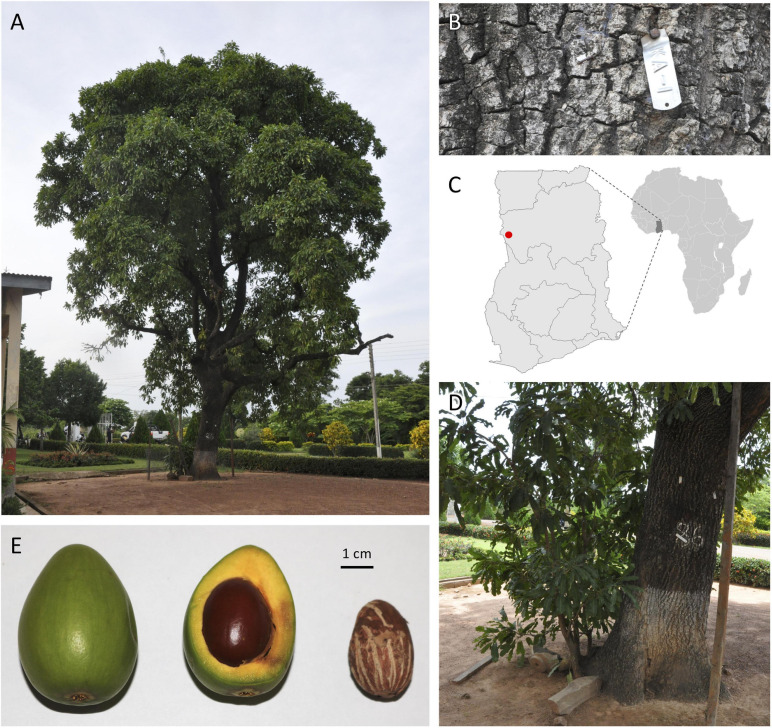
*Vitellaria paradoxa* reference accession ‘KA01’ is a giant-type shea tree **(A,B)** found on the CRIG research substation in Bole in northwestern Ghana (**C**, red dot). For such an aged tree, aggressive root-pruning **(D)** was required to stimulate the production of leaf tissue conducive to high-quality gDNA extraction appropriate for long-read sequencing. **(E)** A typical whole fruit (left), fresh cross-section (middle), and dried kernel (right) from this accession.

At its research substation in the town of Bole in northern Ghana, the Cocoa Research Institute of Ghana (CRIG) has developed and maintains a national shea germplasm repository consisting of an *in situ* population of approximately 600 trees that have been evaluated for yield and basic quality traits annually since 1998. Accession ‘KA01’ stands out as the highest yielding genotype in that collection, with a 10-year average annual nut yield of 22 kg and the highest annual yield (60 kg) recorded for any tree among CRIG’s *in situ* and *ex situ* germplasm collections. A monumental tree (19.5 m tall, 1.3 m diameter at breast height) estimated to be 100–150 years old, ‘KA01’ has a spherical canopy shape (14.2 m mean diameter) and produces generally ovoid fruits with an average weight of 31.0 g (Figure 1). On average, the fruit’s fleshy pulp (mesocarp) comprises 58% of the total fruit weight and has a high sugar content (26.3°Brix). The average dry nut weight for ‘KA01’ is 7.1 g, with a kernel weight ranging from 4.6–5.3 g (mean shelling percentage of 75%) and an average butter yield of 45%.

Fully expanded leaves of ‘KA01’ have undulate margins and an average blade length of 17.9 mm, width of 5.1 mm, and petiole length of 9.4 mm. The tree is an early season bearing genotype, meaning it initiates flowers in the month of October and begins dropping its fruits in April, earlier than most trees in and around Bole which are observed to initiate fruit drop in June. Unlike many accessions in CRIG’s collection, ‘KA01’ has been found to be highly amenable to vegetative propagation through both stem cuttings and grafting. Although the accession is a giant type tree and is susceptible to pestalotia leaf spot, a major disease of shea which attacks both seedlings and mature trees (Akrofi and Amoah, 2009), its relatively stable yield and desirable butter characteristics recommend it as a promising breeding parent; thus it has been used extensively in CRIG’s crossing programs to date, including as the male line in a recent nested association mapping (NAM) population. For these reasons, ‘KA01’ was selected as the *V. paradoxa* accession for developing an annotated reference genome for this outcrossing, diploid (2n = 24) species (Miège, 1954; Löve, 1980; Johnson, 1991) within the Sapotaceae.

To enable broadly relevant SNP discovery and genome-assisted breeding in shea, a concerted effort was made to collect foliar tissue from a diverse collection of *V. paradoxa* accessions from across the shea belt. In total, 150 silica-dried leaf samples were received at the genomics laboratory of the African Orphan Crops Consortium (AOCC) at World Agroforestry (CIFOR-ICRAF) in Nairobi, Kenya, for DNA extraction. Following extraction, a subset of 100 accessions was selected to represent a geographically diverse resequencing panel, distributed as follows (west to east): Senegal (5 lines), Côte d’Ivoire (15), Ghana (70), Benin (18), Nigeria (5), Cameroon (4), Chad (5), and Uganda (27 – *V. paradoxa* ssp. *nilotica*). Of these 100 lines, however, skim sequencing library preparation was ultimately successful for only 31 due to the challenges of tissue collection in remote areas and the extraction of high-quality genomic DNA (gDNA) from this species (see Methods), thus reducing the geographical representation of the panel to four countries [Ghana (23), Benin (2), Nigeria (4), and Cameroon (2)] (for details, see Additional File 2: Supplementary Table 1). To supplement the shallow resequencing data of the above panel, gDNA was also extracted from fresh leaf tissue of a shea seedling growing in the Nairobi, Kenya, nursery of World Agroforestry (CIFOR-ICRAF genebank accession ‘ICRAFF 11537’) for deeper Illumina shotgun sequencing (see Variant Analysis). According to genebank records, the origin of the seedling is CIFOR-ICRAF’s shea germplasm collection in Bamako, Mali.

Consistent with the Nagoya Protocol on Access and Benefit Sharing, an instrument of the Convention on Biological Diversity (CBD), shea tissues were in all cases collected by collaborating national program scientists (see section “Acknowledgments”) who were responsible for any necessary collection consent in their respective countries. The tissue samples shipped out of the respective countries were used only for DNA/RNA extraction, in accordance with MTA’s. All DNA and RNA samples were then sent to the United States and South Africa, respectively, for analysis for research purposes only; and this research has no direct commercial application.

### Reference Genome Sequencing and Assembly

#### Extraction of DNA and Sequencing

To induce new shoot and leaf growth conducive to high molecular weight gDNA extraction from the mature reference accession ‘KA01,’ the tree was root pruned in the summer of 2016 (Figure 1D). Three different consignments of young leaves were then collected and shipped to the AOCC Genomics Laboratory for sequential rounds of gDNA extraction, in August 2016 (250 leaves), February 2017 (500 leaves), and February 2018 (500 leaves).

Extracting high-quality gDNA from the mucilage-rich foliar tissue of the shea tree proved to be a serious challenge, so it is worth detailing the successful protocol here. Using a modified CTAB procedure developed at the AOCC Laboratory, 4–6 young leaves (3–5 g) at a time were ground in liquid nitrogen, to which 25 mL of preheated (65^0^C) high salt CTAB buffer (100 mM Tris-Cl, pH 8.0; 20 mM EDTA, pH 8.0; 3 M NaCl; freshly added with 3% PVP, β-mercaptoethanol, and 3% CTAB) were added. The solution was incubated for 45 min in a 65^0^C water bath, then cooled in an ice bath. At this point, half the volume (12.5 ml) of 5M NaCl was added, followed by the addition of one-tenth the volume (2.5 ml) of chilled isopropanol, and the solution gently mixed via swirling. Leaf debris was removed via refrigerated centrifugation (3,500 × *g* at 4°C for 20 min), resulting in the separation of a clear supernatant that was then subjected to two rounds of purification and precipitation.

In the first round, the supernatant was purified by adding an equal volume of dichloromethane, centrifuging as before, then adding the same volume of chloroform:isoamyl alcohol (24:1) and centrifuging again. The purified DNA was precipitated by adding an equal volume of isopropanol and storing at 4°C for 1–3 h. The precipitated DNA was pelletized via centrifugation, washed with 70% ethanol (5–10 mL), dried at room temperature, and then dissolved in 500 μL of TE (10 mM Tris-Cl, pH 8.0;1 mM EDTA, pH 8.0). Following suspension, 10 μL of RNase (10 mg/mL) was added and the solution incubated at room temperature for 1 h. In the second round, an equal volume of chloroform:isoamyl alcohol (24:1) was again added, the solution centrifuged (13,000 × *g* at 4°C for 15 min), and the supernatant separated. The re-purified DNA, now free of residual RNase, was precipitated by adding double the volume of chilled absolute ethanol and storing at –20°C for 1–2 h. The precipitated DNA was pelletized, washed, and dried as before and finally dissolved in a minimum volume of TE (100–150 μL).

The integrity of the suspended DNA was inspected on a 0.8% agarose gel, its optical density ratios were checked using a Nanodrop spectrophotometer (ND-2000, Thermo Fisher Scientific, Waltham, United States), and final quality checking was performed using a Qubit 2.0 (Thermo Fisher Scientific). As they became available, batches of extracted gDNA were sent to the UC Davis Genome Center, where a 20-kb BluePippin kit (PacBio) was used for Single Molecule Real Time (SMRT) library preparation. Ultimately, three separate libraries were prepared and sequenced on a total of 25 SMRT cells on a PacBio Sequel system, using V2 chemistry.

#### Assembly, Phasing, and Scaffolding

The FALCON and FALCON-Unzip toolkits (FALCON-integrate v1.8.2) (Chin et al., 2016) were used for whole genome assembly and phasing. FALCON is a Hierarchical Genome Assembly Process (HGAP) pipeline that generates a genome assembly from long PacBio reads through the following basic steps: (1) Raw read error correction via alignment of subreads; (2) Pre-assembly of long, error-corrected reads; (3) Overlap detection among pre-assembled reads; (4) Overlap filtering; (5) Overlap graph construction; and (6) Graph-based contig construction. After the initial assembly, FALCON-Unzip is used in highly heterozygous species like shea to resolve the distinct haplomes (i.e., unzip the genome) based on patterns of structural variants and associated SNPs (i.e., haplotype blocks). This unzipping process gives rise to a set of so-called primary contigs (the primary assembly) and a set of associated haplotigs (phased variants of primary contig segments spanning regions of high heterozygosity). Complete details of the FALCON assembly parameters used in this study are provided in Additional File 3: Supplementary Text 1. Finally, the Arrow algorithm from the ‘GenomicConsensus’ PacBio package^1^ was used to polish the primary contigs and their associated haplotigs.

High levels of heterozygosity in some genomic regions can lead to the incorrect assignment of haplotigs as distinct primary contigs, thus requiring further polishing and curation of the assembly via the Purge Haplotigs pipeline (Roach et al., 2018). To identify such errors and correctly assign homologous contigs to the haplotig pool, the Purge Haplotigs pipeline first performs a read-depth analysis using BEDTools (Quinlan and Hall, 2010) to flag abnormally low or high coverage contigs as potential chimeras and then performs a BLAST (Camacho et al., 2009) against the entire assembly to identify putative primary contigs exhibiting high homology to one another. During this process, alignment dotplots are produced, and these are manually screened to break likely chimeras, define the final set of primary contigs as the reference sequence, and assign residual syntenic contigs as haplotigs. Complete details of the Purge Haplotigs process are provided in Additional File 3: Supplementary Text 2.

To extend haplotype phasing to chromosome scale, the Purge Haplotigs output was integrated with chromosome conformation capture (Hi-C) data via FALCON-Phase (Kronenberg et al., 2021). The final result of the FALCON-Phase workflow are pairs of scaffolds (phase 0 and phase 1), such that all phase blocks along the scaffolds originate from the same parental haplotype. As part of this workflow, Proximity-Guided Assembly was performed using Phase Genomics’ Proximo^TM^ Hi-C analysis (Burton et al., 2013). Tissue processing, chromatin isolation, library preparation, sequencing, and Hi-C analysis were performed by Phase Genomics (Seattle, WA, United States), including a final stage of manual curation of the Hi-C assembly using JuiceBox (Dudchenko et al., 2018). Due to their slightly superior assembly statistics (total length, length N50, etc.), the phase 0 scaffolds were selected as the reference for all downstream analysis.

#### Assessment of Assembly Quality

Quality of the final curated assembly was assessed using QUAST (Gurevich et al., 2013), and assembly completeness was evaluated using the set of 1,614 core Embryophta plant genes in BUSCO v4 (Simão et al., 2015). To identify and purge contaminant contigs, the final assembly was aligned using BLAST to the following databases of possible contaminants: plasmid DNA (cpDNA and mtDNA) from angiosperms, the human genome (GRCh38.p7), the *Escherichia coli* genome (CP017100.1), and 16S and 18S rRNAs. The rRNA database was created using the SILVA project (Quast et al., 2013), and the others were created via sampling from Genbank.

### Genome Annotation

#### Transcriptome Assembly

Total RNA was extracted from nine different tissues: flowers (buds and mature), fruits (skin and pulp), and dried seeds collected from mature reference accession ‘KA01’; and roots, stems, and leaves (buds and mature) collected from juvenile accession ‘ICRAFF 11537.’ All samples were finely chopped, stored, and transported in RNA*later* (Thermo Fisher Scientific, Catalog #AM7021) to the CIFOR-ICRAF AOCC Genomics Laboratory, where total RNA was extracted using the Zymo Quick-RNA^TM^ Plant Miniprep kit (Catalog #R2024). In addition to the quantification and quality assessment described previously for the gDNA, RIN values of the purified RNA were estimated using BioAnalyzer 2100 (Agilent, Santa Clara, CA, United States). A pooled RNA-seq library, equimolar by tissue type, was prepared using Illumina TruSeq^®^ RNA Library Prep Kits and sequenced via 100 bp paired-end (PE) reads on an Illumina HiSeq 2500 at the Agricultural Research Council Lab in Pretoria, South Africa. The same pool was used to generate a complementary set of long-read RNAseq data via a single-end run of Oxford Nanopore Technology (ONT) at the University of Dundee, United Kingdom.

For the Illumina reads, CASAVA-processed raw sequences were error-corrected using the software BFC v1.0 (Li, 2015), following recommendations from the Oyster River Protocol For Transcriptome Assembly (MacManes, 2016). Error-corrected reads were then processed to remove Illumina adapters and trimmed to remove low quality reads (Phred ≤ 5) using Trimmomatic v.0.33 (Bolger et al., 2014). All post-processed reads from the nine tissues were pooled *in silico*, and a pure Illumina transcriptome was assembled using Trinity (reference-guided *de novo* assembly) (Haas et al., 2013). The ONT data set was corrected using proovreads (Hackl et al., 2014), generating a set of corrected ONT reads. Because the ONT platform generates long sequences from single molecules, the sequencing products are considered to be the transcripts themselves. By merging the corrected ONT data set with the pure Illumina assembly and removing redundancy using Vsearch (Rognes et al., 2016), a third ‘hybrid’ assembly was also generated. The relative qualities of the three assemblies were evaluated via basic summary statistics and quality metrics, and the completeness of each was assessed using the set of 1,614 core Embryophyta plant genes in BUSCO v4 (Simão et al., 2015). Finally, the results of the three assemblies were concatenated and the quality and completeness of the integrated RNA assembly was re-evaluated.

#### Gene Prediction

RepeatModeler v2.0 (Flynn et al., 2020) was used to identify the repeat families in the genome assembly, and RepeatMasker v4.1 (Smit and Hubley, 2013) was used to discover and classify repeats based on the custom repeat libraries from RepeatModeler v2.0. Gene models were predicted via a combination of *ab initio* prediction, homology search, and RNA-aided annotation. For *ab initio* gene prediction, model training based on the alignment of short RNAseq data was accomplished using BRAKER2 (Brůna et al., 2021). For homology prediction, protein sequences from three related species within the order Ericales [*Actinidia chinensis* (kiwifruit), *Rhododendron simsii* (indoor azalea), and *Camellia sinensis* (tea plant)] were used as query sequences to search the reference genome using TBLASTN (*e*-value < 1e-5); and the regions mapped by these query sequences were compared using Exonerate (Slater and Birney, 2005). For transcriptome-guided annotation, the integrated RNA assembly was used as input to the Program to Assemble Spliced Alignments (PASA) pipeline. Finally, EVidenceModeler v1.1.1 (Haas et al., 2008) was used to integrate the results of the three prediction strategies above, based on different evidentiary weights. TransDecoder, a companion software of the Trinity platform, was used to predict open reading frames; and putative gene function was identified using InterProScan in conjunction with different databases, including PFAM, Gene3D, PANTHER, CDD, SUPERFAMILY, ProSite, and GO. Further functional annotation of the predicted genes was obtained by aligning the protein sequences against those in public protein databases and the UniProt database using BLASTP (*e*-value < 1e−5).

### Phylogenetic and Whole Genome Duplication Analyses

#### Phylogenetic Analysis

To retrieve the evolutionary history of the Ericales, we chose five species [*A. chinensis*, *R. simsii*, *C. sinensis*, *Primula vulgaris* (primrose), and *V. paradoxa*], representing five Ericales families, as well as five other species representing extended diversity within the Asterid [*Daucus carota* (wild carrot), *Solanum lycopersicum* (tomato), and *Camptotheca acuminata* (tree of life)] and out-group Rosid [*Vitis vinifera* (grape) and *Arabidopsis thaliana*] clades. The amino acid sequences of all proteins were downloaded from PLAZA (Van Bel et al., 2018) and NCBI, and OrthoFinder v1.1.4 (Emms and Kelly, 2019) was used to identify orthologous groups (all-versus-all BLASTP with an *e*-value < 1e−05). Single-copy orthologs were extracted from the clustering results; and pre-alignment homology filtering was done using PREQUAL (Whelan et al., 2018), followed by multiple sequence alignment of the masked amino acid sequences using MAFFT (Katoh and Standley, 2013) with default parameters. Finally, protein sequence alignments were transformed into codon alignments. The resulting codon alignments from all single-copy orthologs were then concatenated into one representative supergene for species phylogenetic analysis. A maximum likelihood phylogenetic tree of single-copy codon alignments from shea and the nine other angiosperms was constructed using IQ-TREE with GTR + G model and 1,000 bootstrap replicates. Divergence times between the eight Asterid species and the two Rosid outgroups were estimated using MCMCtree from the PAML package (Yang, 2007) under the GTR with Gamma (GTR + G) model, calibrating the results with (1) the crown node of Ericales (89.8 Mya), (2) the Asterid – Rosid divergence (116–126 Mya), and (3) the *S. lycopersicum* – *D. carota* divergence (95–106 Mya). MCMCTree was used to obtain 10,000 samples from the posterior, sampling every 150 iterations after a burn in of 500,000 iterations; and convergence was verified via independent runs.

#### Whole Genome Duplication Analysis

*K*_S_ distribution analysis was performed using the wgd package (Zwaenepoel and Van de Peer, 2019), with the paranome (entire collection of duplicated genes) obtained via ‘wgd mcl’ using all-against-all BlastP and MCL clustering. The *K*_S_ distribution of shea was then constructed using ‘wgd ksd’ with default settings [i.e., using MAFFT for multiple sequence alignment, codeml for maximum likelihood estimation of pairwise synonymous distances, and FastTree (Price et al., 2009) for inferring phylogenetic trees used in the node weighting procedure]. So-called “anchors” or “anchor pairs” (i.e., paralogous genes located within broader regions of collinearity/synteny in the genome) were obtained using i-ADHoRe (Simillion et al., 2008; Proost et al., 2012), employing the default settings in ‘wgd syn.’ Within the shea genome, the MCScanX toolkit (Wang et al., 2012) was used to identify collinear blocks; and TBtools (Chen et al., 2020) was used to visualize the results via a circos plot. MCScanX was also used to perform comparative pair-wise genomic analyses among *V. paradoxa*, *C. sinensis*, and *V. vinifera* (Tang et al., 2008).

### Analysis of Fatty Acid (FA) Biosynthesis Homologs

To identify genes related to pathways that govern FA biosynthesis in shea, the lipid metabolic pathway was downloaded from the *A. thaliana* acyl-lipid metabolism database^2^ and full-length protein sequences of the implicated lipid metabolic genes were downloaded from the TAIR database^3^. Additional relevant protein sequences from *A. thaliana* and *Theobroma cacao* (cocoa tree) were downloaded from PLAZA^4^. FA biosynthesis homologs in shea and *T. cacao* were identified via BLASTP searches against the protein sequences of fetched *A. thaliana* lipid metabolic genes. Candidate FA biosynthesis genes in *A. thaliana*, *T. cacao*, and *V. paradoxa* were aligned using MAFFT (Katoh and Standley, 2013) and concatenated for phylogenetic analysis. Maximum likelihood gene trees based on protein sequences were constructed using IQ-TREE with the GTR + G model and 1,000 bootstrap replicates (Nguyen et al., 2015). Finally, microsynteny plots were generated using MCScan-Jcvi (Tang et al., 2008) as a means of probing the origins of those FA biosynthesis genes exhibiting relatively high copy number in shea.

### Variant Analysis

Three sequencing datasets were used to call SNPs independently. The first consists of a total of 92 Gb of 150-bp reads of the reference accession ‘KA01,’ generated by 10x Genomics, with an average 137x coverage of the genome. Long Ranger v2.2.2 was used, in conjunction with GATK v4.0.3.0 (McKenna et al., 2010), to map the 10x reads and call SNPs, which were then phased and screened by 10x specific filters. The second sequencing dataset contains 22 Gb of 150-bp Illumina shotgun reads of shea accession ‘ICRAFF 11537,’ with average 34x coverage of the shea genome. The third data set consists of skim resequencing of 31 individual genotypes using Illumina, with an average of 1.8x coverage of mapped reads across the population. Both Illumina data sets were mapped using BWA v0.7.16a and called for SNPs using GATK v4.1.6.0. Details of all applied filters for each dataset are provided in Additional File 2: Supplementary Table 2. Of all the SNPs called, only those that were shared across at least two datasets (10x data of ‘KA01,’ Illumina shotgun data of ‘ICRAFF 11537,’ and the resequencing panel) were advanced as high-confidence SNPs for functional analysis using the SnpEff pipeline (Cingolani et al., 2012).

### SNP Selection and Diversity Analysis

To facilitate the translation of this work into ready-to-use tools of practical value for shea improvement programs, two different SNP panels were developed from the full set of high-confidence SNPs described above (see Variant analysis). The first panel, intended for use in genome-wide association studies (GWAS), genomic selection (GS) programs, germplasm characterization, linkage map development, trait mapping, and the like, requires a set of broadly applicable SNPs evenly distributed across the genome. To accomplish this, the genome was divided into 5 kb contiguous bins and the SNP with highest MAF chosen to represent each bin. The second panel, intended specifically for the targeted characterization of FA biosynthesis genes, requires a set of broadly applicable SNPs within (or in tight linkage with) those genes. To accomplish this, two SNPs were selected per identified FA biosynthesis homolog, with priority given to SNPs of high MAF and secondarily to those with a putative effect on gene function, based on the SnpEff analysis. For both the GWAS and FA panels, selected SNPs were required to be bordered on either side by at least 50 bp of conserved sequence (i.e., no known nearby sequence variation) to facilitate targeted primer and/or bait design. To maximize informativeness and the chance of broad applicability of the panels to other collections of *V. paradoxa* germplasm, all selected SNPs were required to be present in all genotypic states (homozygous major allele, heterozygous, and homozygous minor allele) within the resequencing panel.

## Results

### An Annotated, Chromosome-Scale Reference Genome for Shea Tree

Approximately 162.6 Gb of sequence data from reference accession ‘KA01’ was generated from 25 PacBio Single Molecule Real Time (SMRT) cells (V2 chemistry on Sequel), with an average read length of 8,142 bp and a read length N50 of 13,514 bp (Additional File 1: Supplementary Table 3). The FALCON-Unzip pipeline resulted in a 784.3 Mb assembly consisting of 1,215 putative primary contigs with a contig length N50 of 2.03 Mbp (Table 1). Further curation of the primary contigs, in the form of chimera breaking and cryptic haplotig identification (see section “Materials and Methods”), resulted in a final 667.3 Mb assembly consisting of 594 primary contigs with a contig length N50 of 2.44 Mbp (Table 1). Associated with these primary contigs is a corresponding set of 2,517 phased haplotigs (contig length N50 = 376.0 kb), spanning 42.3% of the primary contig space.

**TABLE 1:** Summary statistics of the *V. paradoxa* accession ‘KA01’ genome assembly, by stage.

Variables	FALCON-Unzip		Purge Haplotigs^a^		Hi-C scaffolding
	Primary	Haplotigs	Primary	Haplotigs	
Number of contigs	1,215	2,682	594	2,517	12
Total length (Mbp)	784.26	507.73	667.29	282.00^b^	658.73
Longest (Mbp)	7.62	1.96	7.62	1.96	82.82
Shortest (bp)	21,126	277	277	277	37.28
>100 kbp (%)	831 (68.4)	1,350 (50.3)	468 (78.1)	1,406 (55.9)	14 (100)
>1 Mbp (%)	236 (19.4)	29 (1.1)	224 (37.4)	29 (1.1)	14 (100)
Mean length (Mbp)	0.64	0.18	1.11	0.20	55.89
N50 length (Mbp)	2.03	0.37	2.44	0.38	57.51
GC content (%)	32.96	33.03	32.94	33.01	32.95

*^a^After application of the Purge Haplotigs pipeline and manual contig curation (i.e., chimera breaking and haplotig re-assignment).*

*^b^Total length of primary contig space to which the haplotigs align.*

The primary contigs from the final assembly were placed into chromosome-level scaffolds (i.e., pseudo-molecules) on the basis of three-dimensional proximity information obtained via chromosome conformation capture (Hi-C) analysis (Burton et al., 2013; Bickhart et al., 2017). Of the 594 primary contigs, 495 (combined length of 658.7 Mb; contig N50 of 2.50 Mbp) successfully assembled into 12 pseudo-molecules, as shown in the Hi-C heatmap (Additional File 1: Supplementary Figure 1). The emergence of 12 distinct Hi-C guided pseudo-molecules lends support to previous cytological studies that placed the chromosome number of *V. paradoxa* at 2n = 24 (Miège, 1954; Löve, 1980; Johnson, 1991). The remaining 99 contigs (combined length of 8.56 Mbp, contig N50 of 221.0 kbp) were designated as unscaffolded contigs. Detailed summary statistics of the 12 pseudo-molecules and 99 unscaffolded contigs comprising the 667.2 Mb *V. paradoxa* accession ‘KA01’ reference assembly are reported in Table 2.

**TABLE 2:** Summary statistics of the ‘KA01’ reference genome, by chromosome.

Chr	Length (Mbp)	No. of contigs	Contig length N50 (Mbp)	No. of genes	No. of SNPs	Mean distance between SNPs (bp)
Chr 01	80,731,948	42	2.89	4,208	413,405	195
Chr 02	74,439,616	57	2.39	4,682	392,583	189
Chr 03	57,704,473	44	3.07	3,137	314,591	183
Chr 04	59,651,551	42	2.86	3,283	308,006	193
Chr 05	59,580,608	51	2.15	3,312	321,960	185
Chr 06	49,210,429	36	2.35	3,131	285,150	172
Chr 07	55,380,075	48	2.03	3,200	266,400	207
Chr 08	52,298,408	46	2.09	3,270	273,406	191
Chr 09	47,443,901	42	2.05	2,504	254,725	186
Chr 10	46,597,090	30	2.42	2,596	245,885	189
Chr 11	38,413,107	27	3.13	2,478	212,268	180
Chr 12	37,276,254	30	2.55	2,239	190,581	195
Unscaffolded	8,558,030	99	0.22	465	9,997	–

The RNAseq libraries generated 47.02 Gb of Illumina data (∼376 million PE reads) and 0.56 Gb of ONT reads (778,913 reads of variable lengths; length N50 = 886 bp). The three RNA assemblies, namely the pure Illumina Trinity assembly, the pure Oxford Nanopore (ONT) assembly, and the Vsearch-mediated hybrid of the two, were ultimately integrated, providing 118,065 transcripts to support RNA prediction, of which GMAP was able to align 99.98% to the reference genome. In total, 46,868 gene models (including splice variants) were obtained with the PASA pipeline, of which 42,368 had open reading frames predicted by TransDecoder (for details, see Additional File 2: Supplementary Table 4).

**FIGURE 2:**
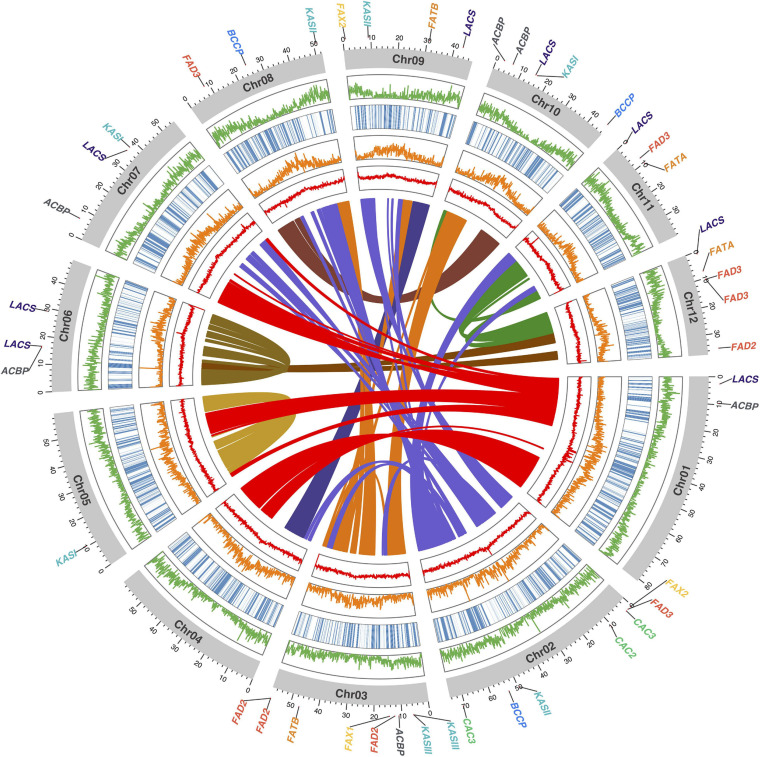
Circos plot of the shea genome, with links between syntenic regions indicated (center). Moving from the center outward, the four circumferential plots per chromosome report mean %GC content (red), number of transposable elements (orange), haplotig coverage (blue heatmap), and number of coding gene models (orange), per 100 kb bin. The coordinate axes track the physical lengths of the chromosomes (Mbp), to which the locations of the 45 fatty acid (FA) biosynthesis homologs identified in this study are anchored.

In terms of structural annotation, transposable elements (TEs) were found to account for approximately 60% of the 658.7 Mbp ‘KA01’ reference genome, with roughly 17% of those TEs being long terminal repeat (LTR) elements (Additional File 2: Supplementary Table 5). As summarized in Table 3, after masking repeat elements, 38,505 coding gene models were found. On average, protein-coding genes in shea are 4,831 bp long and contain 4.4 exons. BUSCO analysis of the predicted protein sequences against the Embryophyta database10 showed that 87.8% of the BUSCO genes were found as complete genes, with an additional 8.2% of genes represented in fragmented form. In summary, 80.4% of the BUSCO genes were present as single copy genes and 7.5% were found in multiple copies. Only 4.0% of the BUSCO gene set was found missing (Additional File 2: Supplementary Table 4). A visualization of the final *V. paradoxa* genome assembly is provided in Figure 2, along with genome-wide trends in GC content, TE density, haplotig coverage, and gene density.

**TABLE 3:** Annotation statistics and completeness assessment of the ‘KA01’ reference genome.

***Annotation statistics***	

No. protein coding genes	38,505
Mean gene length (bp)	4,831
Mean CDS length (bp)	987
Mean no. exons per gene	4.39
Mean exon length (bp)	225
Mean intron length (bp)	1,134

***Completeness assessment^a^***	

Complete BUSCOs	1,417 (87.8%)
Single-copy	1,296 (80.3%)
Duplicated	121 (7.5%)
Fragmented	133 (8.2%)
Missing	64 (4.0%)

*^a^BUSCO v4.0.2; 1,614 Embryophyta genes.*

### A Shared Whole Genome Duplication Event in the Ericales

Within the shea genome, a total of 9,800 anchors (i.e., pairs of paralagous genes in duplicated, collinear regions) were found (Figure 2, center). Nearly all collinear blocks on chromosomes 5 and 6 exhibit intrachromosomal linkages and little collinearity with other linkage groups, implying that these two chromosomes may be fusions of the original subgenomes following an ancient whole-genome duplication (WGD) event.

**FIGURE 3:**
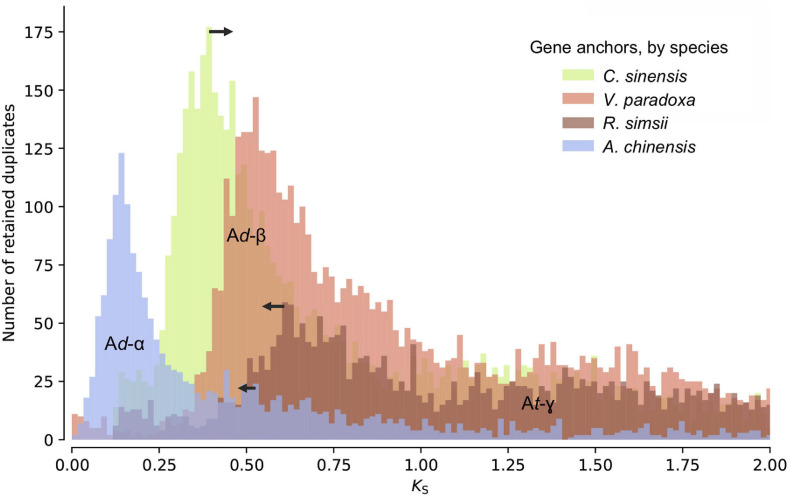
K_S_ age distributions for paralogs of four species within the order Ericales: shea, *Camellia sinensis* (tea plant), *Rhododendron simsii*, and *Actinidia chinensis* (kiwifruit). The blue peak represents the recent WGD event in kiwifruit (A*d*-α). The arrows represent adjustments to synonymous substitution rates based on relative rate tests (see text for details), suggesting a convergence of peaks at a likely shared WGD event in the Ericales (A*d*-β). The well-established ancient WGD event (At-γ) that pre-dates the Asterid-Rosid divergence is also indicated.

Distributions of synonymous substitutions per synonymous sites (*K*_S_) for anchor pairs retained in collinear regions reveal signature peaks at *K*_S_ ≈ 0.6 for a whole-genome duplication (WGD) event in the shea genome, *K*_S_ ≈ 0.45 for a WGD event in *C. sinensis*, and *K*_S_ ≈ 0.73 for a WGD event in *R. simsii* (Figure 3). Apart from these three WGD events, *K*_S_ distributions also provide evidence for two WGDs in *A. chinensis*, a lineage reported to have gone through three distinct WGD events (A*d*-α,17.7–26.5 Mya; A*d*-β, 61.9–73.7 Mya; and A*t*-γ, 150.4–159.3 Mya) (Wu et al., 2019).

**FIGURE 4:**
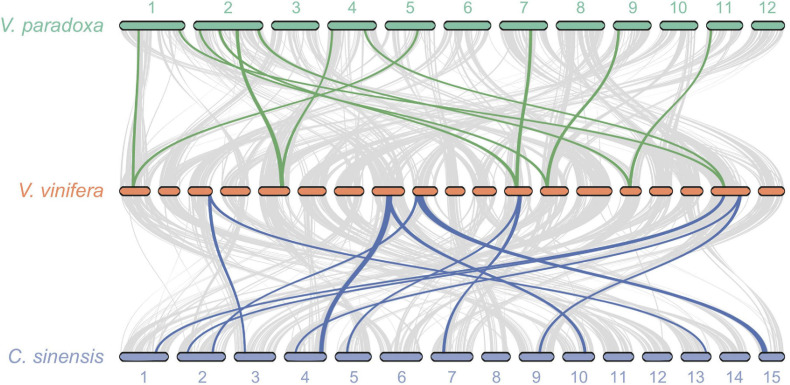
Syntenic relationships between *Vitis vinifera* (grape) and both shea (top) and the tea plant *Camellia sinensis* (bottom) reveal widespread patterns of gene duplication, providing further evidence of WGD events in the two latter species subsequent to their divergence from the Rosids. The gray lines connect all regions of synteny between species (i.e., the background collinearity). Between shea and grape, a total of 15 2:1 syntenic blocks are detected, containing 1,035 genes. Of those, an illustrative subset of six blocks are highlighted (green lines). Similarly, of the 21 2:1 syntenic blocks (1,309 genes) between tea plant and grape, six are highlighted (blue lines).

Because no additional WGD events have been reported for well-studied *V. vinifera* since the ancient A*t*-γ WGD shared by most, if not all, eudicots, we undertook pairwise syntenic analyses between grape and shea, as well as between grape and tea plant. These analyses revealed widespread 2:1 syntenic relationships between shea and grape as well as between tea plant and grape (Figure 4), providing additional evidence for a WGD event in both shea and tea plant since A*t*-γ. Similar results were found by Yang et al. (2020) for *R. simsii*, a related species in the Ericales. Such evidence, particularly in combination with the clear pattern of synteny between putative homoeologous scaffolds (Figure 2), strongly suggest that all three species experienced second WGD events after the common A*t*-γ event.

To determine whether these second WGD events were independent or instead shared by shea, tea plant, and *R. simsii*, and what their relationship might be to those observed in kiwifruit (A*d*-α and A*d*-β), additional evidence was brought to bear. First, as indicated in the K_S_ distributions (Figure 3), the most recent WGD event (A*d*-α) in the kiwifruit genome occurred well after its divergence from shea, supporting the hypothesis that A*d*-α occurred independently in *Actinidia*. Second, a recent study by Wu et al. (2019) indicates a shared A*d*-β event in the Ericales; and related work on the tea plant genome concluded that the A*d*-β event occurred prior to the divergence between tea plant and kiwifruit (Xia et al., 2017). To determine whether the WGD event in the shea genome is lineage-specific or shared with those reported in kiwifruit, tea plant, and *R. simsii* (i.e., A*d*-β) (Xia et al., 2017; Wu et al., 2019; Yang et al., 2020), we compared one-to-one orthologous K_S_ distributions between shea and each of those species (see Additional File 1: Supplementary Figure 2). The K_S_ peak value representing the divergence between shea and *C. sinensis* is smaller (i.e., ‘younger’) than those representing the divergences between shea and either *R. simsii* or kiwifruit, each exhibiting almost equal *K*_S_ values. In other words, the same speciation event is found to correspond to slightly different *K*_S_ peak values, likely due to different substitution rates for different species.

When comparing the one-to-one orthologous *K*_S_ distribution between grape and shea with those between grape and four other species within the order Ericales [tea plant, kiwifruit, *R. simsii*, and primrose], we similarly observe different *K*_S_ peak values for the same speciation event (i.e., the Asterid-Rosid divergence) (see Additional File 1: Supplementary Figure 3). These different peaks again suggest different synonymous substitution rates among the species, with primrose (not included in the anchor pair analysis due to the lower quality of its assembly) as the fastest, *R. simsii* and kiwifruit as relatively slower, and tea plant as the slowest, likely resulting in an overestimation of the divergence time between shea and *R. simsii*/kiwifruit while an underestimation for the divergence time between shea and tea plant. In light of this, relative rate tests were employed to adjust the synonymous substitution rates (see arrows in Figure 3), providing further support of the idea that the WGD event in the shea genome occurred at the same time as the one in tea plant, *R. simsii*, and kiwifruit, prior to their divergence.

**FIGURE 5:**
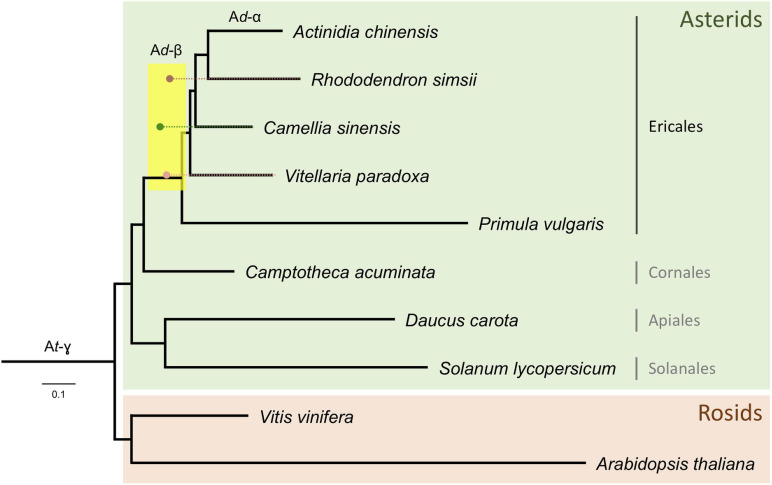
Phylogenetic tree of the Ericales, rooted with Rosids, with branch lengths in *K*_S_ units. Corrected *K*_S_ ages (see text for details) of WGD events identified from anchor pairs in *R. simsii*, *C. sinensis*, and shea are indicated by brown, green, and pink dots, respectively. A likely shared WGD event in the Ericales (A*d*-β) is spanned by the yellow rectangle.

In conclusion, upon reconstructing the phylogeny of the Ericales and other clades with branch lengths in *K*_S_ units (i.e., a *K*_S_ tree) using the codeml package from the PAML software, it can be seen that the *K*_S_ peak values for the WGD events in shea, tea plant, and *R. simsii* reconcile at the same node (Figure 5), corresponding to a shared WGD event (A*d*-β). Application of the Whale method (Zwaenepoel and Van de Peer, 2019) for gene tree/species tree reconciliation also resulted in additional support for this conclusion of a shared A*d*-β event among shea, tea plant, *R. simsii*, and kiwifruit (and likely *P. vulgaris*) within the Ericales clade (see Additional File 2: Supplementary Table 6). Considering previous estimates, this shared WGD likely occurred 65 – 90 Mya (Xia et al., 2017; Wu et al., 2019; Yang et al., 2020).

### Fatty Acid Biosynthesis Genes in Shea

Shea butter is becoming an increasingly valuable ingredient in cosmetics, but its primary commercial value resides in its serving as a high quality cocoa butter equivalent (CBE) in the international chocolate industry. The fact that shea butter contains higher levels of C18 fatty acids than cocoa butter (Di Vincenzo et al., 2005; Jahurul et al., 2013), particularly in the form of stearic acid, suggests that *V. paradoxa* may harbor either a greater number and/or more efficient homologs of FA biosynthesis genes than those found in *T. cacao*. Recent work based on transcriptome data revealed that the shea tree genome indeed encodes a relatively high number of lipid biosynthesis genes (Wei et al., 2019). In this study, the ‘KA01’ reference genome allowed the first whole-genome investigation of the suite of FA biosynthesis genes in shea.

Great strides have been made in characterizing the network of FA biosynthesis enzymes and the underlying genes that encode them. The activity of acetyl-CoA carboxylase (ACC), the key enzyme that catalyzes the necessary and rate-limiting step of *de novo* FA synthesis, is composed of biotin carboxyl carrier protein (BCCP), biotin carboxylase (BC), and the α- and β-subunits of carboxyltransferase (CT) (Li-Beisson et al., 2013). Previous studies in *A. thaliana* showed that BCCP is encoded by two genes (*CAC1A*/*BCCP1* and *CAC1B*/*BCCP2*), while BC, CTα, and CTβ are encoded by single genes (*CAC2*, *CAC3* and *accD*, respectively). Of the genes mentioned, *accD* is the only FA-related gene residing in the plastome; so it was not considered in this analysis. In addition to the ACC enzyme, other key catalytic components of FA biosynthesis belong to the 3-ketoacyl-ACP synthase (KAS) family, members of which include KASIII for the initial combination of acetyl-CoA and malonyl-ACP, KASI for sequential elongation to 16:0-ACP, and KASII (or FAB1) for final extension to 18:0-ACP (see He et al., 2020). KASII activity is therefore a major determinant of the ratio of C18 to C16 FAs in plant cells (Pidkowich et al., 2007). Finally, there are the two sets of fatty acid desaturases (FADs) that catalyze subsequent desaturation steps in the endoplasmic reticulum (ER), namely *FAD2* and *FAD3*. In addition to the genes directly involved in FA biosynthesis, there are others involved in the transmembrane transport of FAs from the plastid to the ER, including acyl-CoA binding proteins (ACBPs), the *FATTY ACID EXPORT* (*FAX*) gene, and various Long Chain Acyl-CoA Synthetases (LACS) (Li et al., 2016).

Using the 30 known FA biosynthesis genes in *A. thaliana* as queries (see Additional File 2: Supplementary Table 7), a search for their homologs in both *T. cacao* and shea tree resulted in the identification of 35 and 45 genes, respectively, representing 8 gene families. The copy numbers, by gene, are shown in Table 4; and their locations within the shea genome are indicated in Figure 2. Based on copy number, there indeed appears to be more genes associated with FA biosynthesis in shea tree (45) than in either *A. thaliana* (30) or *T. cacao* (35). Ketoacyl-ACP synthase (KAS) gene copies are particularly more numerous in shea (9) compared to *T. cacao* (6), which may contribute to the generally higher lipid content of shea versus cocoa butter. Interestingly, the copy numbers of the *FAD2*, *FAD3*, and *LACS* genes are higher than those found by Wei et al. (2019) based on transcriptome data (4, 5, and 8 vs. 2, 2, and 5, respectively).

**TABLE 4:** Summary table of gene copy numbers of enzymes involved in fatty acid biosynthesis in *Arabidopsis thaliana*, *Theobroma cacao*, and *Vitellaria paradoxa*, using 30 *A. thaliana* genes as queries.

		Gene copy number		
Enzyme		*A. thaliana*	*T. cacao*	*V. paradoxa*
BCCP	Homomeric Acetyl-CoA Carboxylase BCCP subunit	2	3	3
CAC2	Homomeric Acetyl-CoA Carboxylase BC subunit	1	1	1
CAC3	Homomeric Acetyl-CoA Carboxylase alpha-CT subunit	1	2	2
KAS I	Ketoacyl-ACP synthase I	1	2	4
KAS II	Ketoacyl-ACP synthase II	1	3	3
KAS III	Ketoacyl-ACP synthase III	1	1	2
FATA	Acyl-ACP Thioesterase Fat A	2	1	2
FATB	Acyl-ACP Thioesterase Fat B	1	1	2
FAD2	ER Oleate Desaturase	1	2	4
FAD3	ER Linoleate Desaturase	1	3	5
FAX1	Fatty acid exporter 1	1	1	1
FAX2	Fatty acid exporter 2	1	1	2
FAX4	Fatty acid exporter 4	1	1	0
LACS	Long Chain Acyl-CoA Synthetase	9	7	8
ACBP	Acyl-CoA-binding protein	6	6	6

**FIGURE 6:**
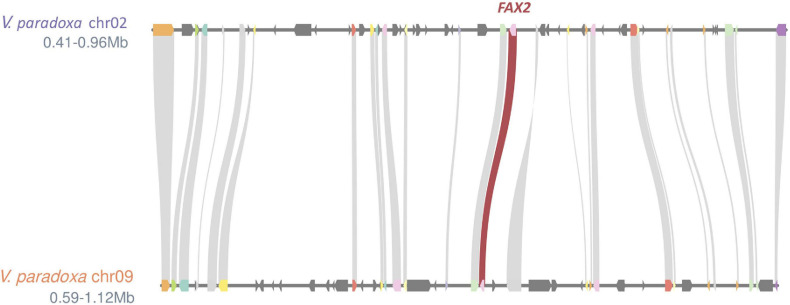
Microsynteny plot showing ∼0.5 Mb regions exhibiting strong synteny (26 gene models) between shea Chromosomes 2 and 9. The existence the two copies of *FAX2* within these larger syntenic neighborhoods suggests that shea’s relatively high copy number of this FA biosynthesis gene likely can be traced to a large-scale duplication event like WGD. Microsynteny plots for *FATB*, *FAD2*, *FAD3*, and (as counterpoint) *KASIII* can be found in Additional File 1: Supplementary Figures 4–7.

Based on the genomewide assessment of synteny presented in Figure 2, four of the FA biosynthesis genes exhibiting higher copy number in shea than in either *A. thaliana* or *T. cacao* appeared to reside within larger syntenic blocks. For each of those genes (*FATB*, *FAD2*, *FAD3*, and *FAX2*), subsequent microsynteny analysis confirmed this conclusion (Figure 6; Additional File 1: Supplementary Figures 4–6), suggesting that these copies likely originated in a large-scale event like WGD. Such a signature of large-scale, dispersed duplication for these four genes stands in stark contrast to the clear tandem/local duplication exhibited, for example, by *KASIII* (Additional File 1: Supplementary Figure 7).

**FIGURE 7:**
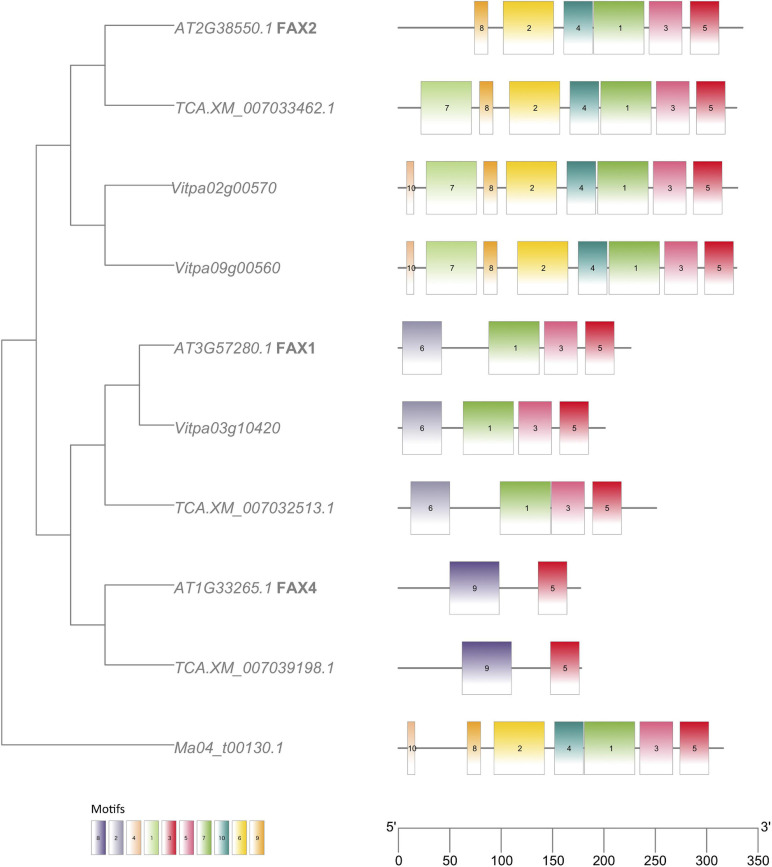
FAX (Fatty acid exporter) gene tree with MEME motifs, comparing orthologs from *Arabidopsis thaliana* (“AT…”), *Theobroma cacao* (“TCA.XM…”) and *Vitellaria paradoxa* (“Vitpa…”). An ortholog from *Musa acuminata* (“Ma…”) of clade Monocots serves as outgroup. Trees for the other FA biosynthesis genes identified in this study can be found in Additional File 1: Supplementary Figures 8–16.

Analyses of MEME motifs for each gene family (Additional File 1: Supplementary Figures 8–16) reveal expected similarities within subfamilies. In the *FAX* family, the *FAX2* genes in shea and *T. cacao* possess an additional motif absent from the *A. thaliana* genome (Figure 7). Both *FAX2* and *FAX4* are important for seed oil accumulation. Although no *FAX4* homologs were found in the shea genome, two *FAX2* genes were identified, compared to the single *FAX2* gene found in both *A. thaliana* and *T. cacao*.

### A SNP Database for the Shea Community

SNP calling under stringent criteria (see Additional File 2: Supplementary Table 2) resulted in the identification of 7.08, 4.45, and 4.47 million bi-allelic SNPs in the resequencing panel of 31 accessions, within reference accession ‘KA01’ (10x data), and within accession ‘ICRAFF 11537’ (Illumina shotgun data), respectively. Of those, a total of 3,488,957 SNPs were found to be shared by at least two of those datasets (see Additional File 1: Supplementary Figure 17). Considering only those ∼3.5 million high-confidence SNPs, SNP density is observed to be similar across shea’s 12 chromosomes, with an average of 1 SNP every 189 bp (Table 2). SnpEff functional annotation of the set of high-confidence SNPs generated 5.6 million predicted effects, more than 97% of which were assigned to the MODIFIER impact class (i.e., no evidence of impact on function; see Table 5). The remaining effects, associated with 152,676 SNPs, are predicted to have HIGH, MODERATE, or LOW impacts on the functions 26,290 implicated genes, as summarized in Table 5.

**TABLE 5:** Summary of SnpEff functional analysis.

SnpEff impact class^a^	Type	Count	Percent (%)
HIGH		3,010	0.05
	splice_acceptor_variant	387	
	splice_donor_variant	340	
	start_lost	280	
	stop_gained	1582	
	stop_lost	421	
MODERATE	missense_variant	80,281	1.43
LOW		69,385	1.24
	initiator_codon_variant	27	
	splice_region_variant	7,486	
	synonymous_variant	61,872	
MODIFIER		5,453,021	97.28
Total		5,605,697	100.00

*^a^Impact classes according to the SnpEff pipeline: (1) **HIGH:** Disruptive impact on protein function via protein truncation, loss of function, or triggering nonsense mediated decay; (2) **MODERATE:** Non-disruptive but may affect protein efficacy; (3) **LOW:** Unlikely to change protein behavior; (4) **MODIFIER:** Usually variants in non-coding regions, where predictions are difficult or there is no evidence of impact.*

Following the variant selection strategies described above (see section “Materials and Methods”), two different SNP panels were developed for ready use by shea improvement programs. The first, intended to support genome-wide association studies, general germplasm characterization work, linkage map development, genomic selection protocols, trait dissection, and the like, consists of 104,211 SNPs distributed relatively evenly across the reference genome (average spacing of 6,350 bp). The second panel, intended to enable the much more specific task of characterizing the diversity of FA biosynthesis genes within shea, consists of 90 SNPs, two per putative FA biosynthesis homolog identified in this study. Detailed information about the SNPs comprising these two panels can be found in Additional Files 4, 5. For programs interested in developing other targeted SNP panels, details of all ∼3.5 million high-confidence SNPs are also provided (see section “Data Availability Statement”).

## Discussion

Despite the shea tree’s long recognized value as a dominant agroecological species fulfilling vital sociocultural, economic, environmental, and nutritional functions for millions of households across rural SSA, it remains semi-domesticated with virtually no history of systematic genetic improvement. The central contribution of this research is an annotated 656.7 Mb reference genome assembly of *Vitellaria paradoxa*, a necessary resource for future genome-enabled conservation and breeding efforts. Hi-C guided contig assembly resulted in 12 distinct pseudo-molecules, backing previous cytological investigations that placed the chromosome number of *V. paradoxa* at 2n = 24; and reference annotation revealed 38,505 coding gene models with only 4% missing BUSCO genes, suggesting an acceptable level of completeness for a first assembly.

The annotated reference enabled a whole genome duplication (WGD) analysis which revealed not only that shea underwent a WGD event but that this event (A*d*-β) was shared with other important species within the cosmopolitan order Ericales, including *C. sinensis*, *R. simsii*, and *A. chinensis* (and likely *P. vulgaris*). Evidence of the even earlier WGD event prior to the Asterid-Rosid divergence (A*t*-γ; 150.4–159.3 Mya and shared with *V. vinifera*) was also detected. Given that gene duplication in general, regardless of mechanism, presents new opportunities for evolution in plants (see e.g., Dubcovsky and Dvorak, 2007; Magadum et al., 2013), this pair of ancient WGD events in shea bodes well in terms of expanding the allelic space relevant to artificial selection targets.

As a first exploration of this idea, the annotated reference was used to provide a first glimpse into the suite of putative fatty acid (FA) biosynthesis genes in shea. Analysis of 30 well-characterized FA biosynthesis genes from *A. thaliana* resulted in the identification of 45 orthologs in shea, representing 8 gene families. Based on copy numbers, there appears to be more genes associated with FA biosynthesis in shea tree than in *T. cacao* (35). As a family, Ketoacyl-ACP synthase (KAS) genes in particular were found at higher copy number in shea, which may contribute to the generally higher lipid content of shea versus cocoa butter. Also worth noting are shea’s higher copy numbers of ER desaturase genes *FAD2* and *FAD3*, key enzymes for Omega-3 FA biosynthesis and thus of potential relevance not only to shea’s nutritional value but also its quality for use in cosmetics. Microsynteny analysis implicated a large-scale duplication event, perhaps the A*d*-β WGD event described above, in the relatively high copy numbers not only of these ER desaturase genes, but of the *FATB* and *FAX2* genes as well. With the loci governing FA biosynthesis in shea now identified, targeted characterizations of germplasm collections are now possible, a first step toward understanding the role of extant allelic diversity on FA composition. In a long-generation species like shea, such an understanding is required for developing rational strategies for selection, whether within naturally regenerating parkland stands or among breeding lines. Of course, the value of the genomic resources presented here is not limited to FA traits, however important they may be. Combined with accurate phenotyping data from proper germplasm collections (e.g., NAM populations, association mapping panels, etc.), this chromosome-scale annotated genome can facilitate efficient progress on a multitude of traits of importance, including *Pestalotia* resistance, fruit morphology, flowering time, precocity, tocopherol content, tree architecture, amenability to propagation, and tolerances to any number of abiotic/environmental stresses.

In addition to the raw sequence data, assembled transcriptome, and annotated reference genome (see section “Data Availability Statement”), a database of ∼3.5 million physically anchored and functionally annotated SNPs has also been generated and made publicly available as part of this work. Based not only on variation within reference accession ‘KA01,’ including those regions with sufficient heterozygosity for phased assembly (haplotigs), but also on variation detected within a larger diversity panel, the SNP database should prove to be a widely useful resource for shea improvement programs across SSA. To facilitate the adoption and practical use of this resource, two pre-selected panels of high-confidence annotated SNPs have been developed, one (∼100k SNPs) in support of genome-wide genotyping and the second (90 SNPs) specifically targeting putative FA biosynthesis genes. Effectively serving as first generation *in silico* SNP chips, these panels provide a ready starting point for programs seeking to undertake the work of genome-assisted germplasm curation, conservation, trait association mapping, and breeding in shea. For fine-mapping or for strategic targeting of other genes of interest, the full SNP database is also available.

Traditionally, the processes of plant domestication and breeding, not to mention deployment of improved genetics and production systems at scale, have been extremely long-term undertakings, even for the most cooperative and fast-cycling of model, annual species. To suggest such painstaking work for a slow-growing tree species like shea, with generation times of 10–25 years and relatively limited global importance, seems wishful at best. And yet, the recent convergence of a suite of biotechnologies (e.g., affordable dense genotyping, gene editing, etc.), breeding strategies [e.g., tricot analysis (Etten et al., 2019) and breeding seedling orchards (Barnes, 1995)], statistical methods [e.g., genomic selection (Heffner et al., 2009) and landscape GWAS (Lasky et al., 2018)], and engagement tools (e.g., smartphone-enabled citizen science) bring the domestication and breeding of even the shea tree into the realm of possibility. Alone, the high-quality reference genome developed here is by no means sufficient for such work; but it is an important foundational resource capable of serving the shea improvement community for years to come. As it has in so many other crops, a well-annotated genome presents new possibilities and is thus likely to stimulate further research and investment.

## Data Availability Statement

All raw sequence data generated for this study are publicly available as NCBI Bioproject PRJNA729422, including: (1) Raw PacBio (SMRT) gDNA sequence data of reference accession ‘KA01’ (Biosamples SAMN19116134 and SAMN21031866); and (2) Raw RNAseq data of accessions ‘KA01’ and ‘ICRAFF 11537’ (Biosamples SAMN19488939 and SAMN19488940). All analytical results are publicly available from the ORCAE database (https://bioinformatics.psb.ugent.be/orcae), including: (1) The final Hi-C guided genome assembly of *Vitellaria paradoxa* reference accession ‘KA01’; (2) A supplemental file of final ‘KA01’ haplotigs; (3) The final transcriptome assembly; (4) A complete set of gene descriptor files; and (5) All genome annotation files; and 6) A vcf file of all ∼3.5 million high-confidence SNPs.

## Author Contributions

IH, FP, AD, and RJ conceived and initiated the study. FP, J-MB, AM, and MB acquired, managed, and facilitated the movement of all relevant germplasm. PH and AV coordinated all sample processing and library preparation. IH, ATOM, SK, and SS built the reference assembly. IH and ATOM assembled the transcriptome. XM and YV annotated the assembly and performed the analyses for whole genome duplication and FA biosynthesis genes. SC and AV did the variant calling. IH oversaw all aspects of the study and led the manuscript preparation. All the authors revised the manuscript and approved the final version.

## Conflict of Interest

The authors declare that the research was conducted in the absence of any commercial or financial relationships that could be construed as a potential conflict of interest.

## Publisher’s Note

All claims expressed in this article are solely those of the authors and do not necessarily represent those of their affiliated organizations, or those of the publisher, the editors and the reviewers. Any product that may be evaluated in this article, or claim that may be made by its manufacturer, is not guaranteed or endorsed by the publisher.

## Footnotes

1
https://github.com/PacificBiosciences/GenomicConsensus

2
http://aralip.plantbiology.msu.edu/pathways

3
https://www.arabidopsis.org/

4
https://bioinformatics.psb.ugent.be/plaza/

## Supplementary Material

Additional File 1: Supplementary Figure 1 Hi-C post-scaffolding heatmap of the primary contigs of *V. paradoxa* reference accession ‘KA01.’

Additional File 1: Supplementary Figure 2 One-to-one orthologous *K*_S_ distributions between shea and three other species within the order Ericales: tea plant (*Camellia sinensis*), *Rhododendron simsii*, and kiwifruit (*Actinidia chinensis*).

Additional File 1: Supplementary Figure 3 One-to-one orthologous *K*_S_ distribution between grape (*Vitis vinifera*) and shea, compared with those between grape and four other species within the order Ericales [tea plant, kiwifruit, *Rhododendron simsii*, and *Primula vulgaris* (primrose)].

Additional File 1: Supplementary Figures 4–7 Microsynteny plots of the regions containing copies of fatty acid biosynthesis genes *FATB*, *FAD2*, *FAD3*, and *KASIII* genes in KA01.

Additional File 1: Supplementary Figures 8–16 Gene trees with MEME motifs, comparing fatty acid biosynthesis orthologs from *Arabidopsis thaliana*, *Theobroma cacao*, and *Vitellaria paradoxa*. Orthologs from *Musa acuminata* of clade Monocots serve as outgroups.

Additional File 1: Supplementary Figure 17 Venn diagram of SNPs called using the three different data sets in this study.

Additional File 2: Supplementary Table 1 Geographical provenances of the 31 shea tree accessions used in the skim resequencing panel to facilitate variant calling.

Additional File 2: Supplementary Table 2 Filters used for SNP calling within the three datasets in this study.

Additional File 2: Supplementary Table 3 Summary of raw PacBio data obtained for *V. paradoxa* reference accession ‘KA01’ via 25 SMRT cells.

Additional File 2: Supplementary Table 4 Descriptive statistics of the various transcriptome assemblies, including the final “Integrated” assembly used for annotation.

Additional File 2: Supplementary Table 5 Structural annotation summary statistics.

Additional File 2: Supplementary Table 6 *K*_S_ peak values of whole-genome duplication events detected in the Ericales.

Additional File 2: Supplementary Table 7 Summary table of the 30 fatty acid biosynthesis genes from *A. thaliana.*

Additional File 3: Supplementary Text 1 Complete FALCON command log for this study, including all specified assembly parameters.

Additional File 3: Supplementary Text 2 Complete Purge Haplotigs command log for this study, including all specified parameters.

Additional File 4 Details of the 90 SNPs comprising the FA biosynthesis gene SNP panel for *V. paradoxa*.

Additional File 5 Details of the 104,211 SNPs comprising the GWAS SNP panel for *V. paradoxa*.
