# Supplementary material for: Genomic Resources to Guide Improvement of the Shea Tree
Source: Front Plant Sci. 2021 Sep 9;12:720670. doi: 10.3389/fpls.2021.720670 (PMC8459026; doi:10.3389/fpls.2021.720670)
Supplement: Additional File 1: Supplementary Figure 1 — Hi-C post-scaffolding heatmap of the primary contigs of V. paradoxa reference accession ‘KA01.’ [file Data_Sheet_1.PDF]

Genomic resources to guide improvement of the shea tree

Hale *et al.* 2021

Additional File 1: Supplementary Figures

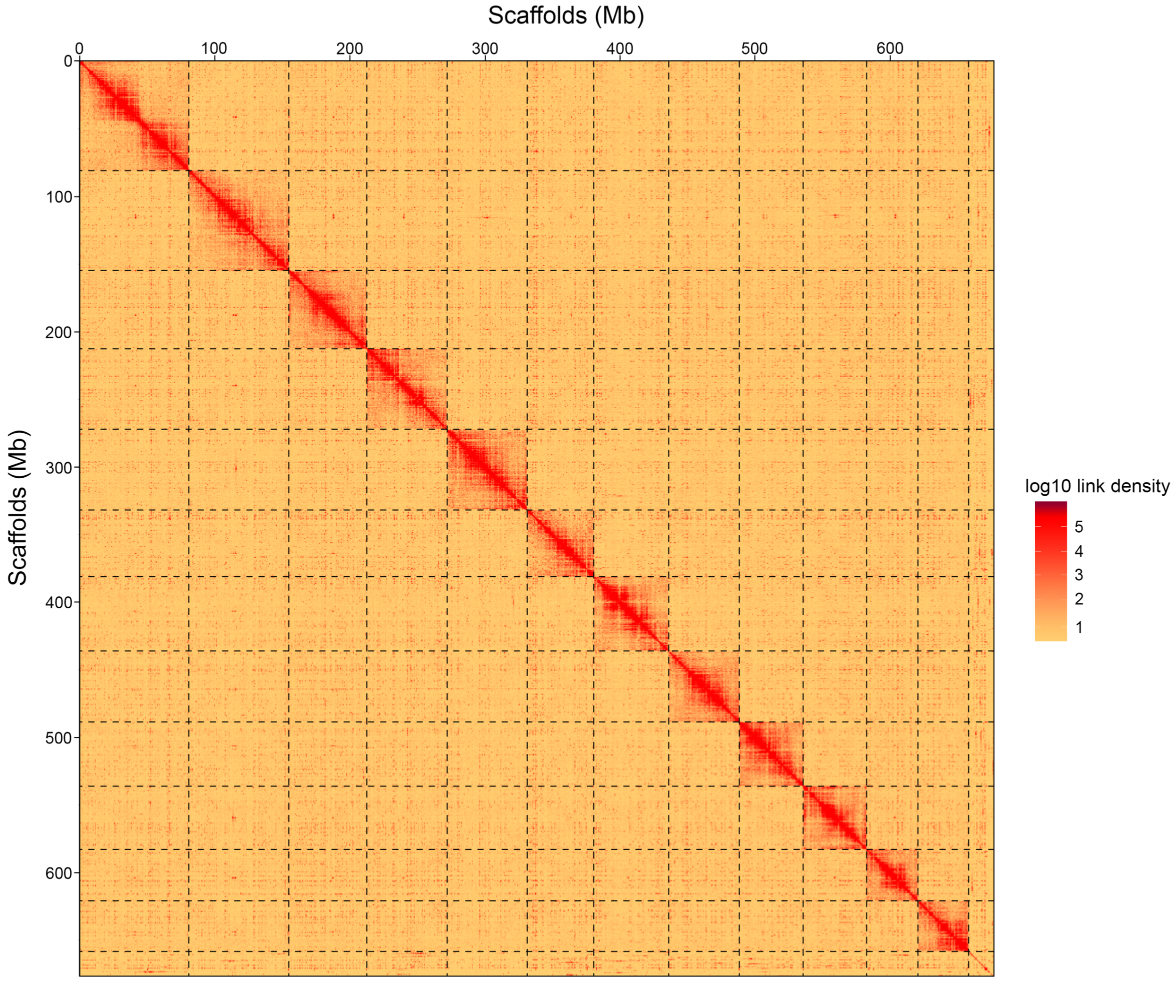

Figure S1. Hi-C post-scaffolding heatmap of the primary contigs of *V. paradoxa* reference accession 'KA01'. Of the 594 primary contigs, 83% (495 contigs, 658.7 Mbp) successfully assembled into 12 pseudo-molecules representing the 12 chromosomes of *V. paradoxa*. Chromosome designations correspond approximately to physical length, with Chromosome 1 being the longest (80.73 Mbp; upper left) and Chromosome 12 being the shortest (37.28 Mbp; lower right). The far lower right block is comprised of the 8.6 Mbp of unscaffolded contigs. See Table 2 for more information.

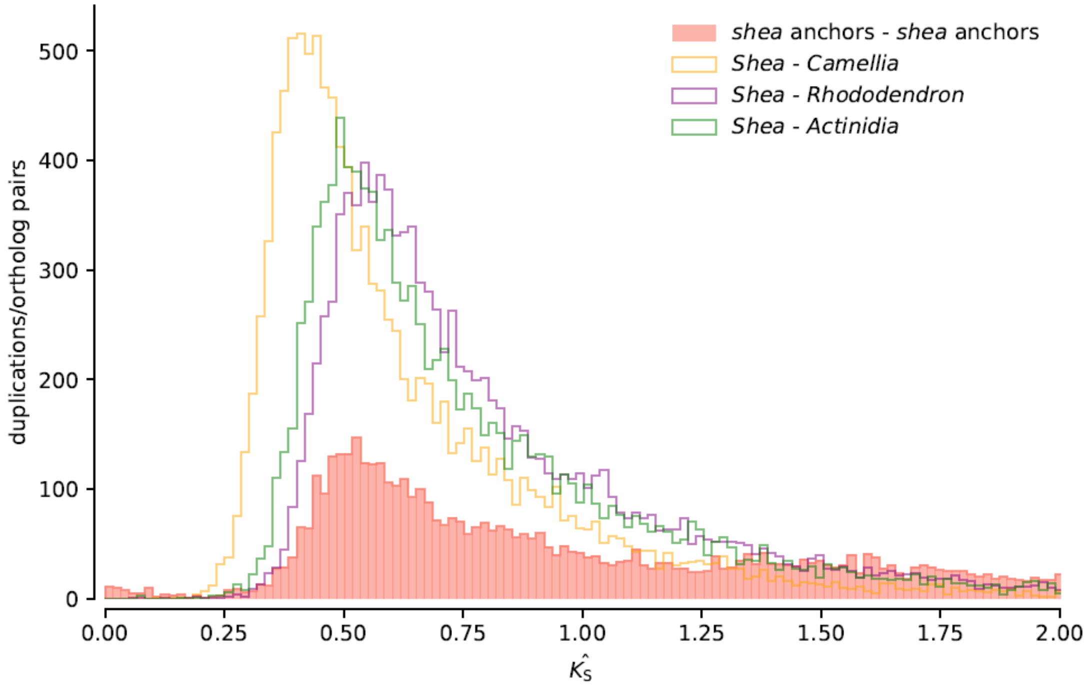

Figure S2. One-to-one orthologous K_S distributions between shea and three other species within the order Ericales: tea plant (*Camelia sinensis*), *Rhododendron simsii*, and kiwifruit (*Actinidia chinensis*). The K_S peak value representing the divergence between shea and *C. sinensis* (~0.4) is smaller than those representing the divergences between shea and either *R. simsii* (~0.55) or *A. chinensis* (~0.5), each exhibiting similar peak K_S values. In other words, the same speciation event (i.e. the ancient A*t*-ɣ WGD event predating the Asterid-Rosid divergence) is found to correspond to slightly different K_S peak values.

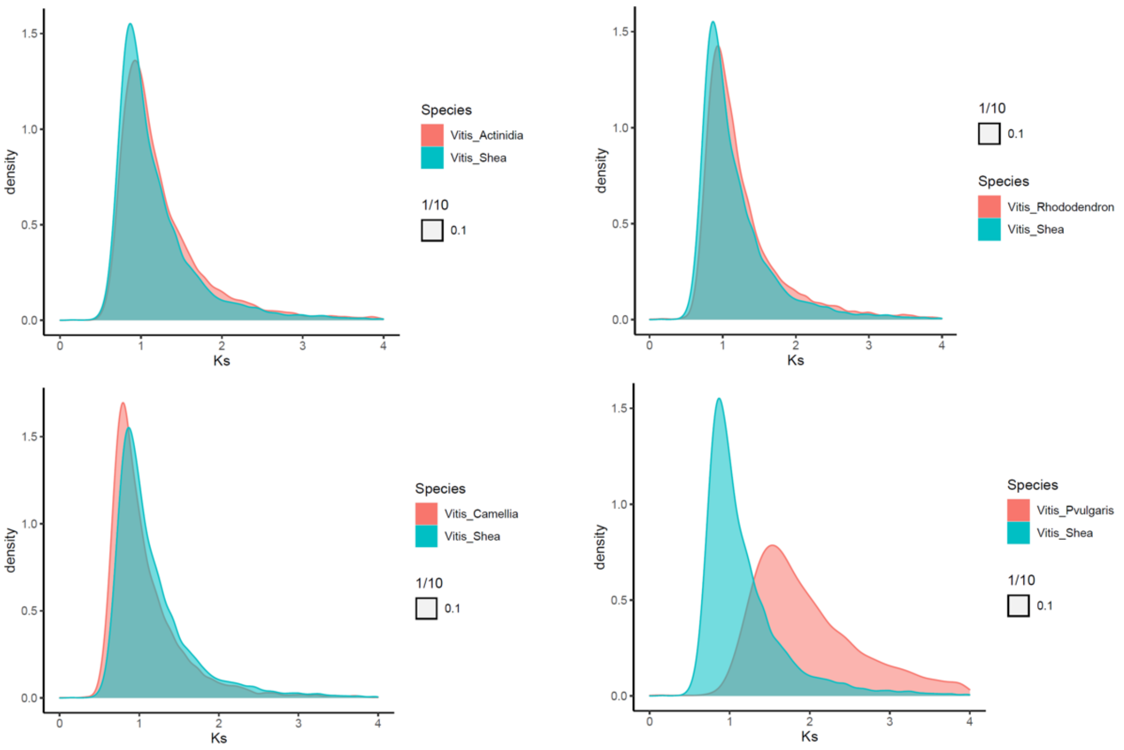

Figure S3. When comparing the one-to-one orthologous K_S distribution between grape (*V. vinifera*) and shea with those between grape and four other species within the order Ericales [tea plant, kiwifruit, *R. simsii*, and *Primula vulgaris* (primrose)], we observe different K_S peaks for the same speciation event (i.e. the ancient A*t*-ɣ WGD event predating the Asterid-Rosid divergence). These different peaks suggest different synonymous substitution rates among the species, with primrose as the fastest, *R. simsii* and kiwifruit as relatively slower, and tea plant as the slowest, likely resulting in an overestimation of the divergence time between shea and *R. simsii*/kiwifruit while an underestimation for the divergence time between shea and tea plant.

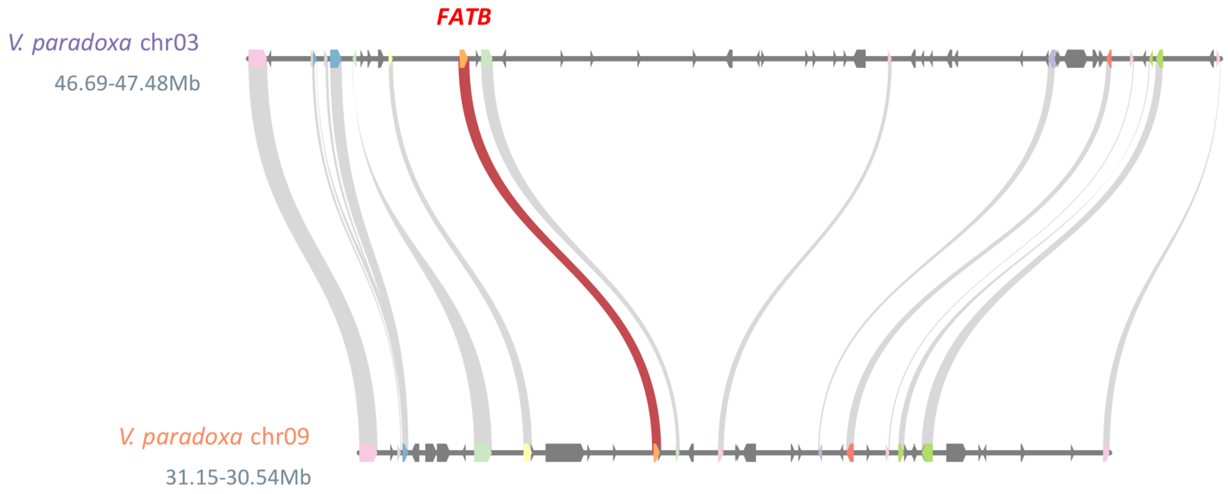

Figure S4. Microsynteny plot showing ~0.6 Mb regions exhibiting strong synteny (14 gene models) between Chromosomes 3 and 9 of reference accession 'KA01'. The two copies of *FATB* within these larger syntenic neighborhoods suggests that shea's relatively high copy number of this FA biosynthesis gene can be traced to a large-scale duplication event like WGD.

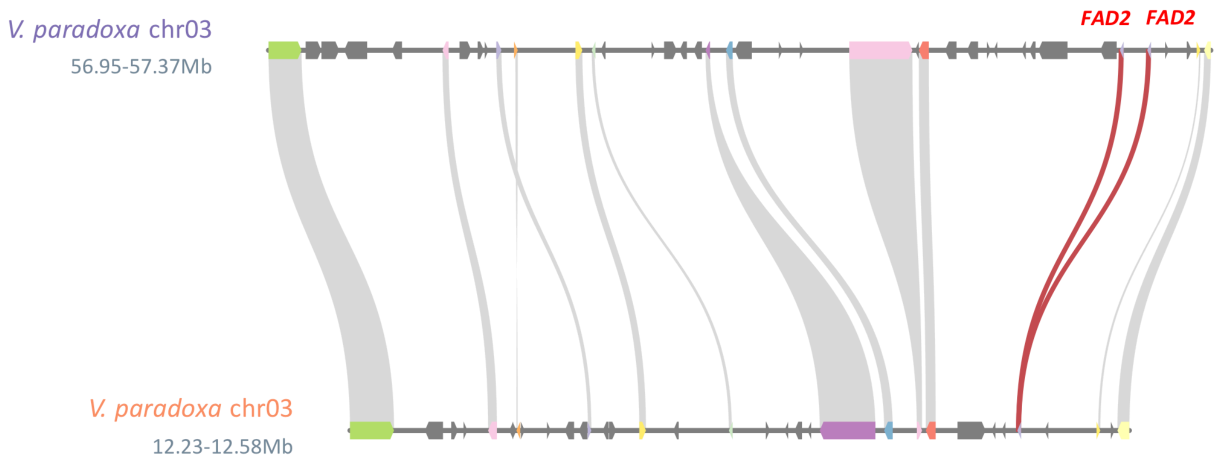

Figure S5. Microsynteny plot showing ~0.35 Mb regions exhibiting strong synteny (13 gene models) between the two arms of Chromosome 3 of reference accession 'KA01'. The copies of *FAD2* within these larger syntenic neighborhoods suggests that shea's relatively high copy number of this FA biosynthesis gene can be traced, at least in part, to a large-scale duplication event like WGD. The tandem/local duplication of *FAD2* suggested by the upper plot served to further increase copy number.

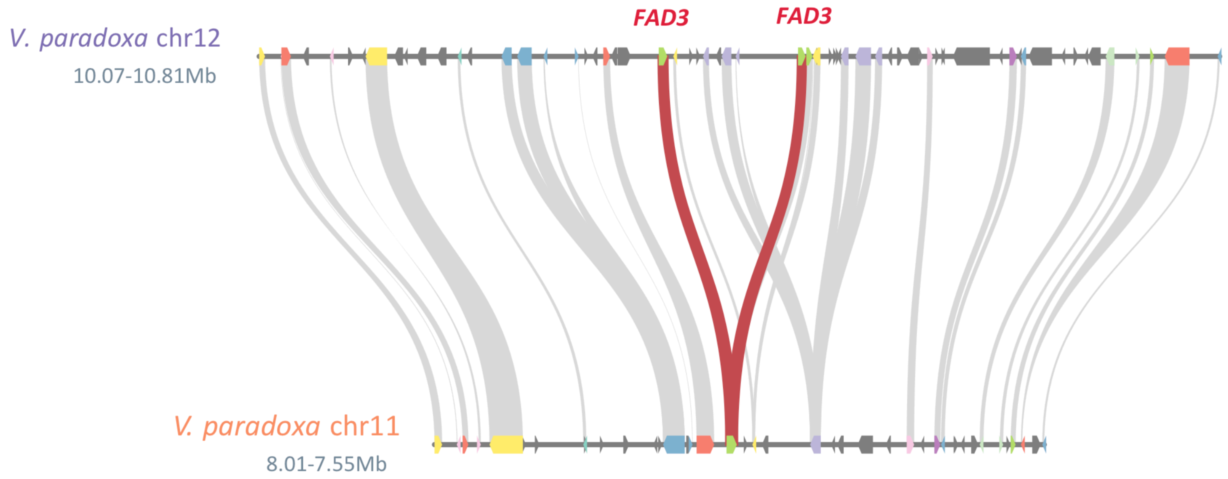

Figure S6. Microsynteny plot showing ~0.6 Mb regions exhibiting strong synteny (20 gene models) between Chromosomes 11 and 12 of reference accession 'KA01'. The copies of *FAD3* within these larger syntenic neighborhoods suggests that shea's relatively high copy number of this FA biosynthesis gene can be traced, at least in part, to a large-scale duplication event like WGD. The tandem/local duplication of *FAD3* and adjacent genes suggested by the upper plot served to further increase copy number.

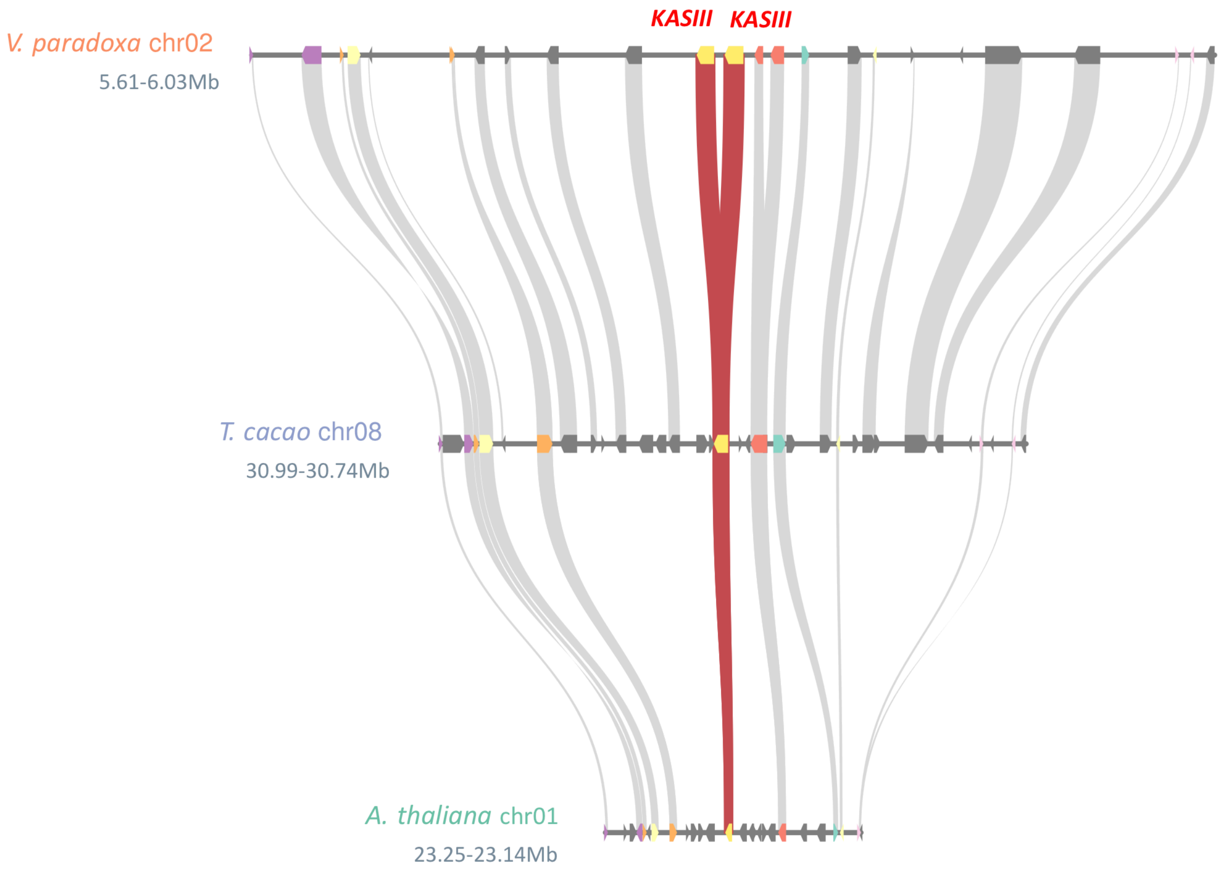

Figure S7. Microsynteny plot showing the *KASIII*-containing region exhibiting strong synteny between shea, *T. cacao*, and *A. thaliana*. In this case, the relatively high copy number of *KASIII* in 'KA01' appears due to a tandem/local duplication rather than a large-scale event like WGD.

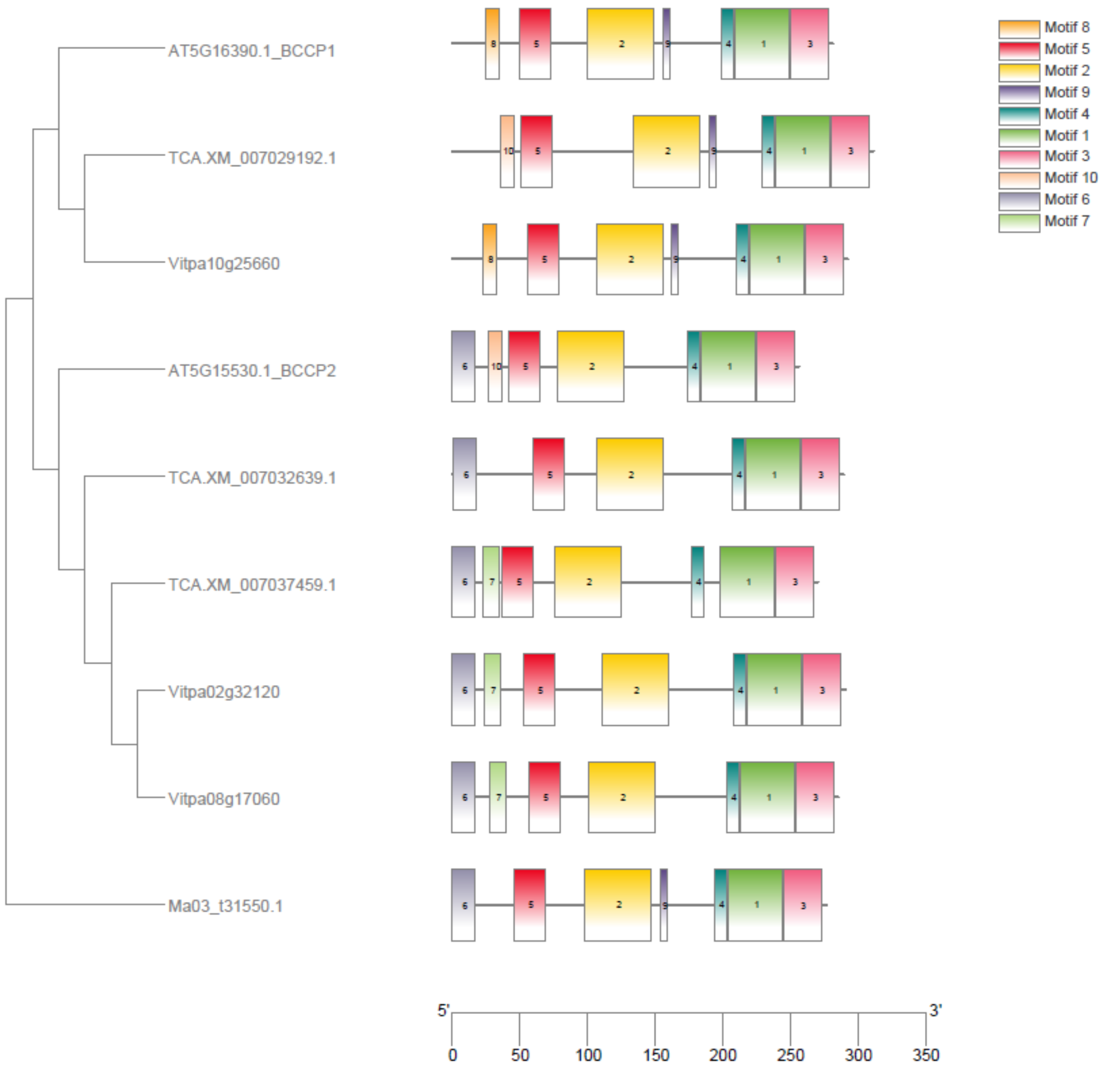

Figure S8. BCCP (Homomeric Acetyl-CoA Carboxylase BCCP subunit) gene tree with MEME motifs, comparing orthologs from *Arabidopsis thaliana* ("AT…"), *Theobroma cacao* ("TCA.XM…") and *Vitellaria paradoxa* ("Vitpa…"). An ortholog from *Musa acuminata* ("Ma…") of clade Monocots serves as outgroup.

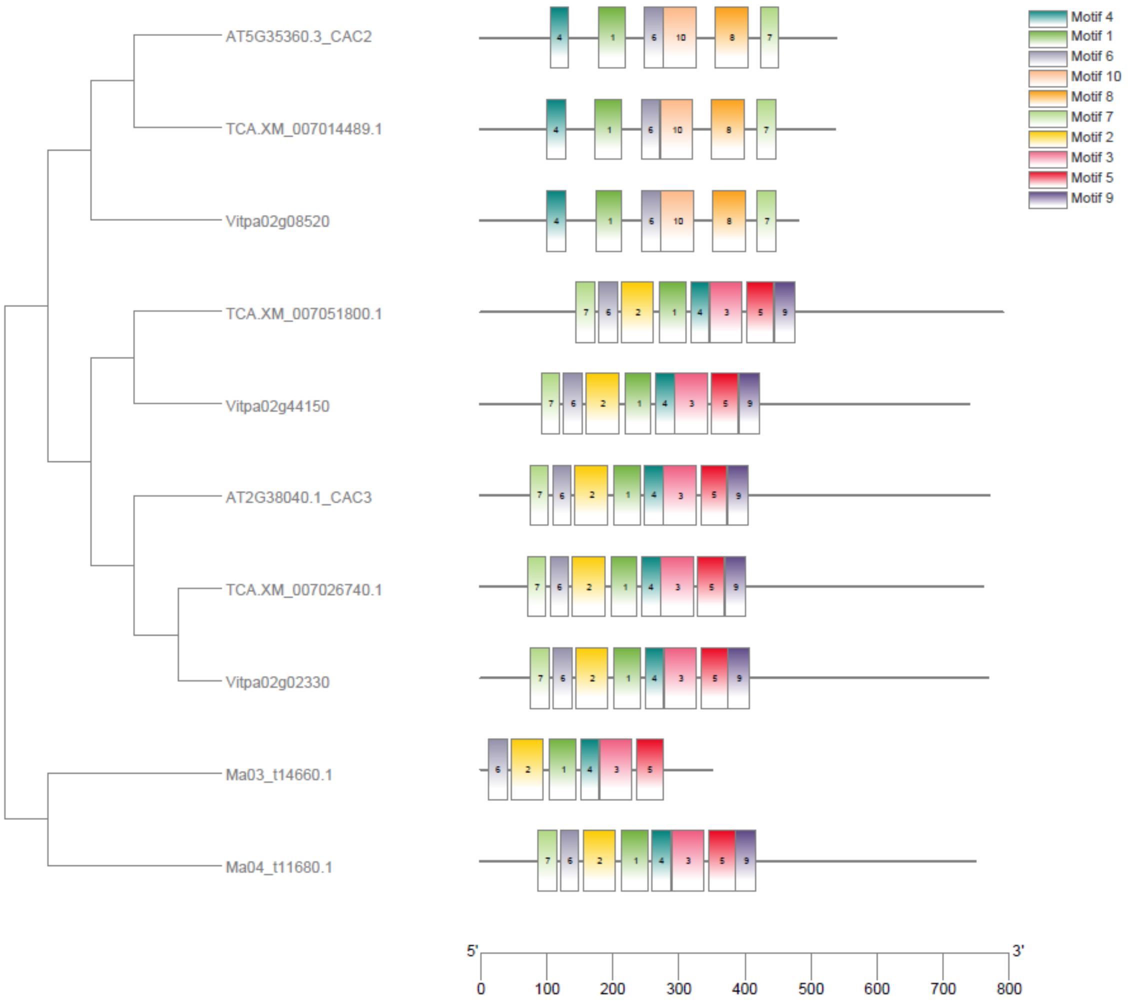

Figure S9. CAC (Homomeric Acetyl-CoA Carboxylase BC subunit) gene tree with MEME motifs, comparing orthologs from *Arabidopsis thaliana* (“AT…”), *Theobroma cacao* (“TCA.XM…”) and *Vitellaria paradoxa* (“Vitpa…”). Orthologs from *Musa acuminata* (“Ma…”) of clade Monocots serve as outgroup.

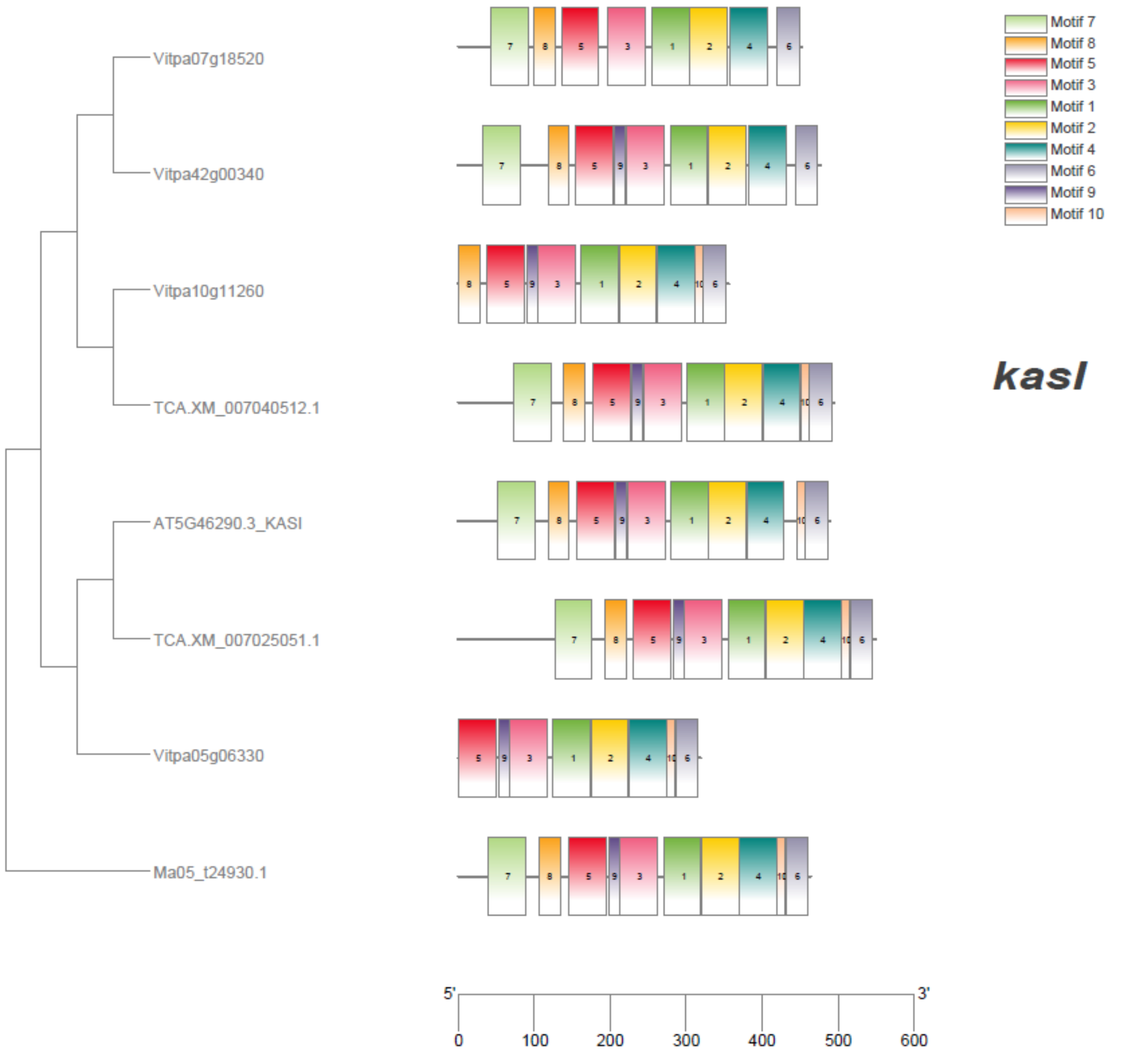

Figure S10. KASI (Ketoacyl-ACP synthase I) gene tree with MEME motifs, comparing orthologs from *Arabidopsis thaliana* ("AT…"), *Theobroma cacao* ("TCA.XM…") and *Vitellaria paradoxa* ("Vitpa…"). An ortholog from *Musa acuminata* ("Ma…") of clade Monocots serves as outgroup.

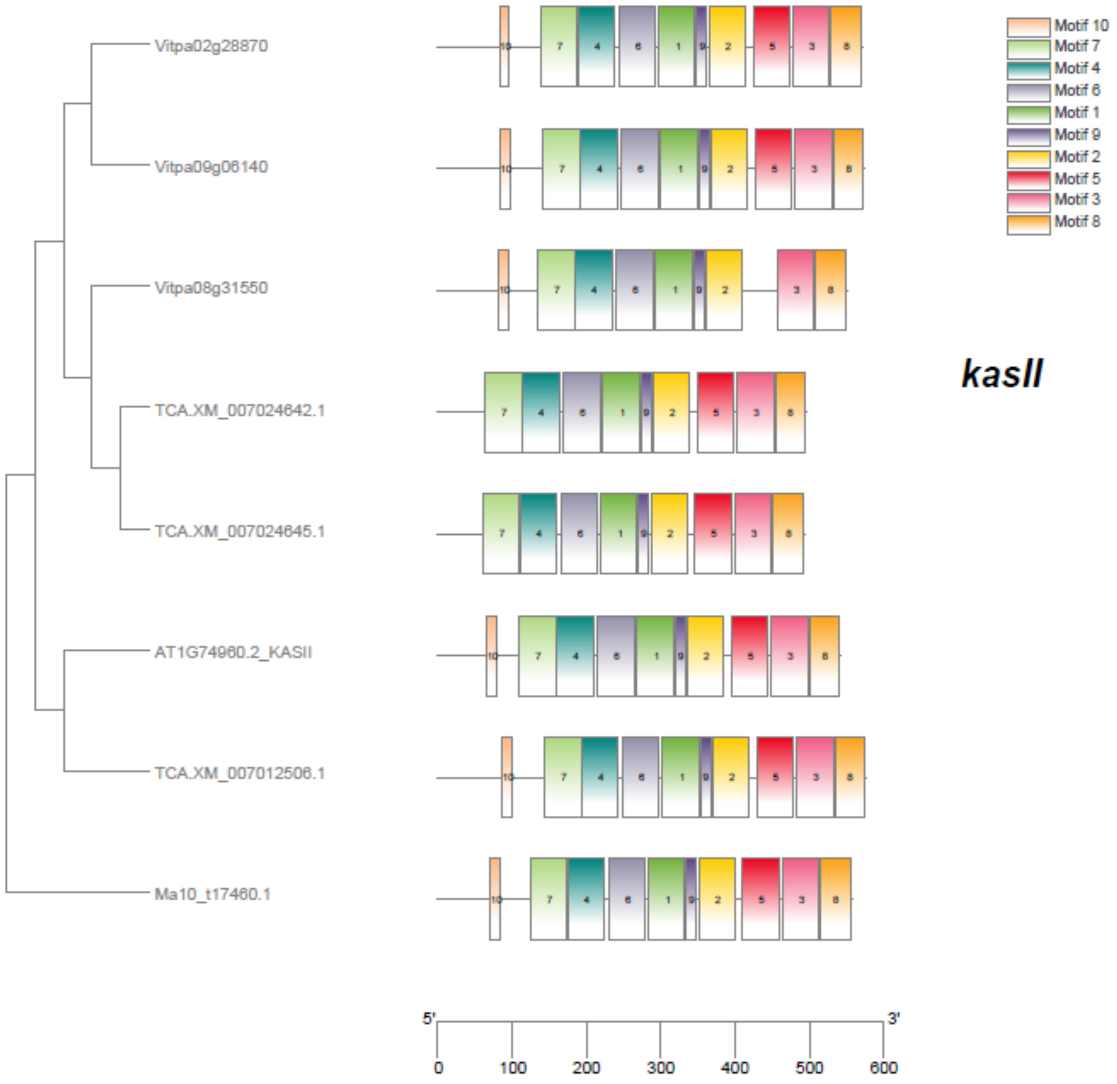

Figure S11. KASII (Ketoacyl-ACP synthase II) gene tree with MEME motifs, comparing orthologs from *Arabidopsis thaliana* ("AT…"), *Theobroma cacao* ("TCA.XM…") and *Vitellaria paradoxa* ("Vitpa…"). An ortholog from *Musa acuminata* ("Ma…") of clade Monocots serves as outgroup.

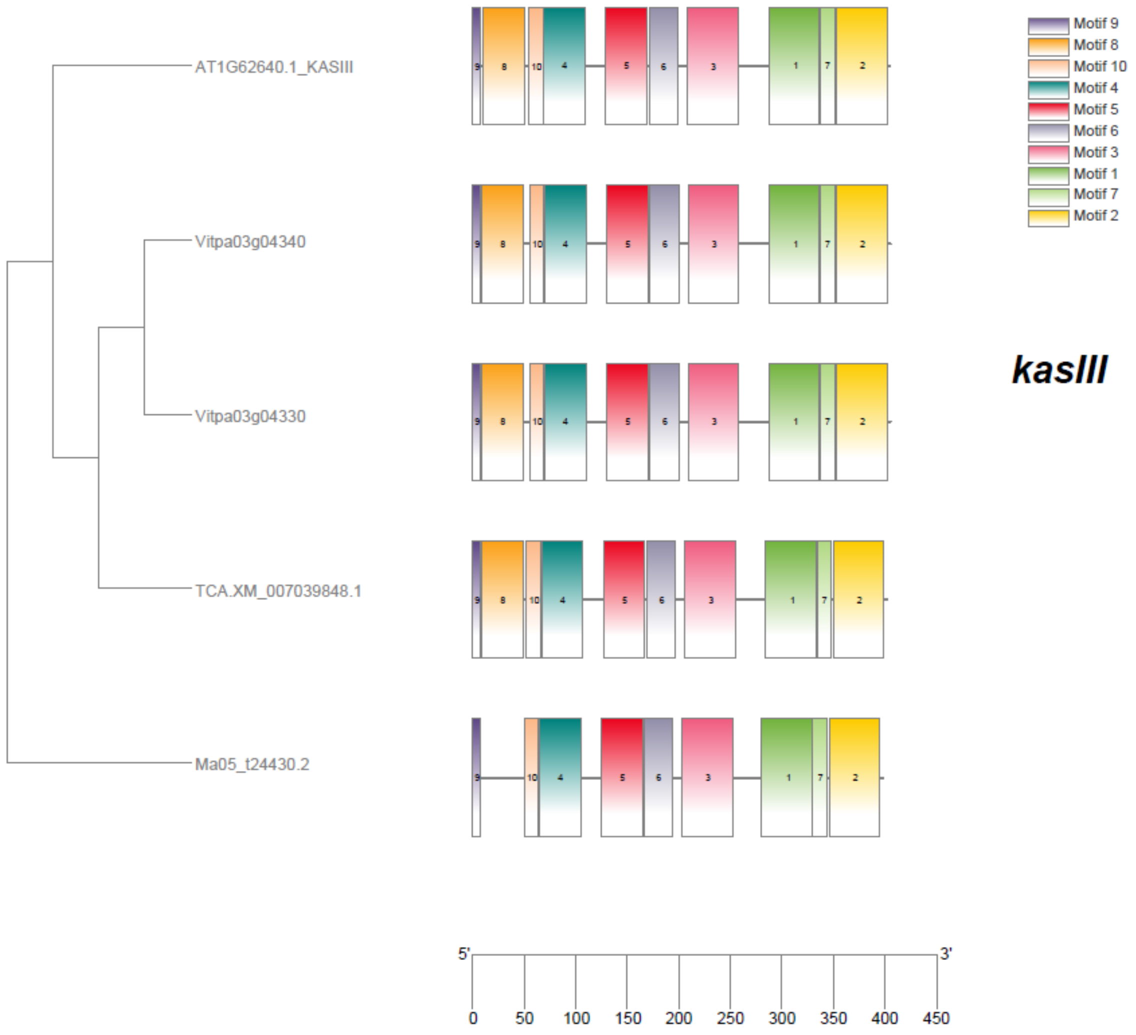

Figure S12. KASIII (Ketoacyl-ACP synthase III) gene tree with MEME motifs, comparing orthologs from *Arabidopsis thaliana* ("AT…"), *Theobroma cacao* ("TCA.XM…") and *Vitellaria paradoxa* ("Vitpa…"). An ortholog from *Musa acuminata* ("Ma…") of clade Monocots serves as outgroup.

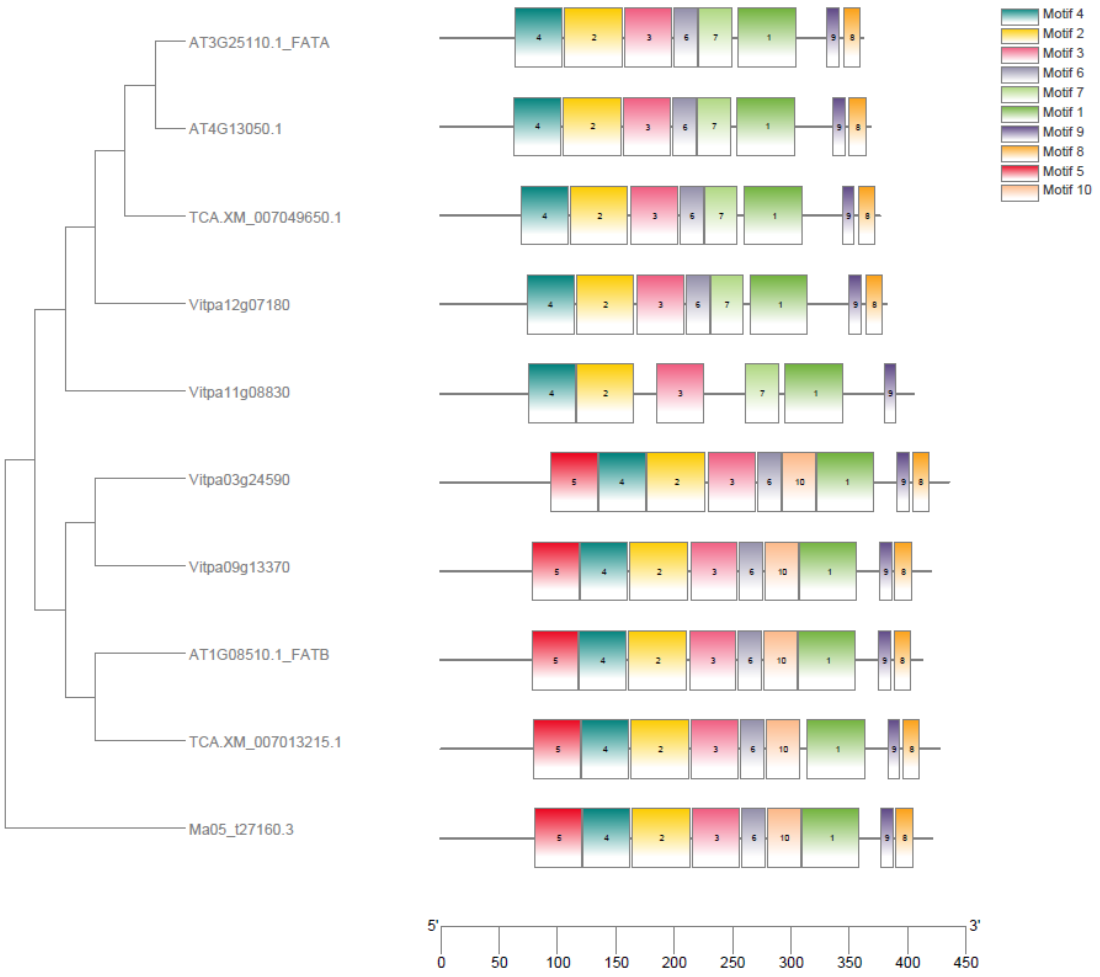

Figure S13. FATA/B (Acyl-ACP Thioesterase Fat A/B) gene tree with MEME motifs, comparing orthologs from *Arabidopsis thaliana* ("AT…"), *Theobroma cacao* ("TCA.XM…") and *Vitellaria paradoxa* ("Vitpa…"). An ortholog from *Musa acuminata* ("Ma…") of clade Monocots serves as outgroup.

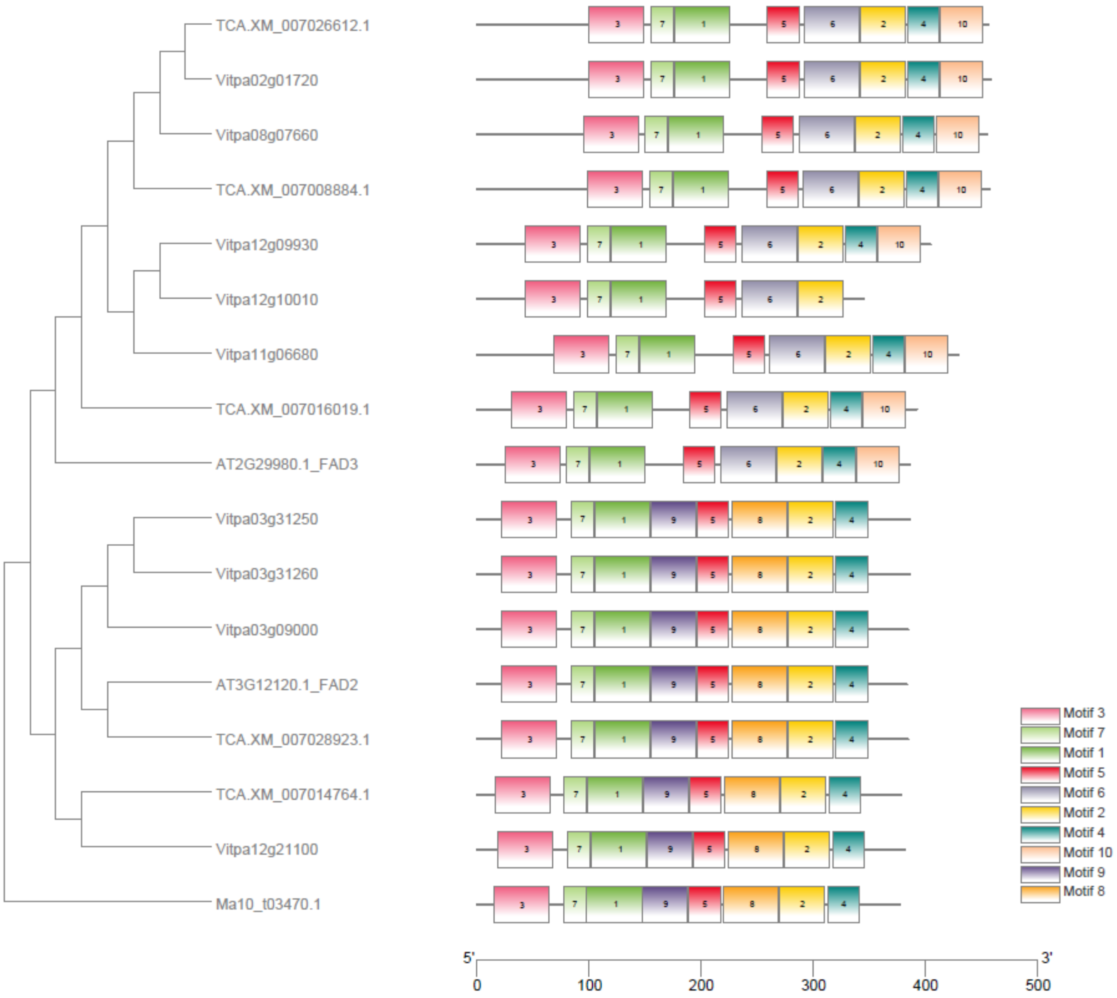

Figure S14. FAD (Fatty acid desaturase) gene tree with MEME motifs, comparing orthologs from *Arabidopsis thaliana* ("AT…"), *Theobroma cacao* ("TCA.XM…") and *Vitellaria paradoxa* ("Vitpa…"). An ortholog from *Musa acuminata* ("Ma…") of clade Monocots serves as outgroup.

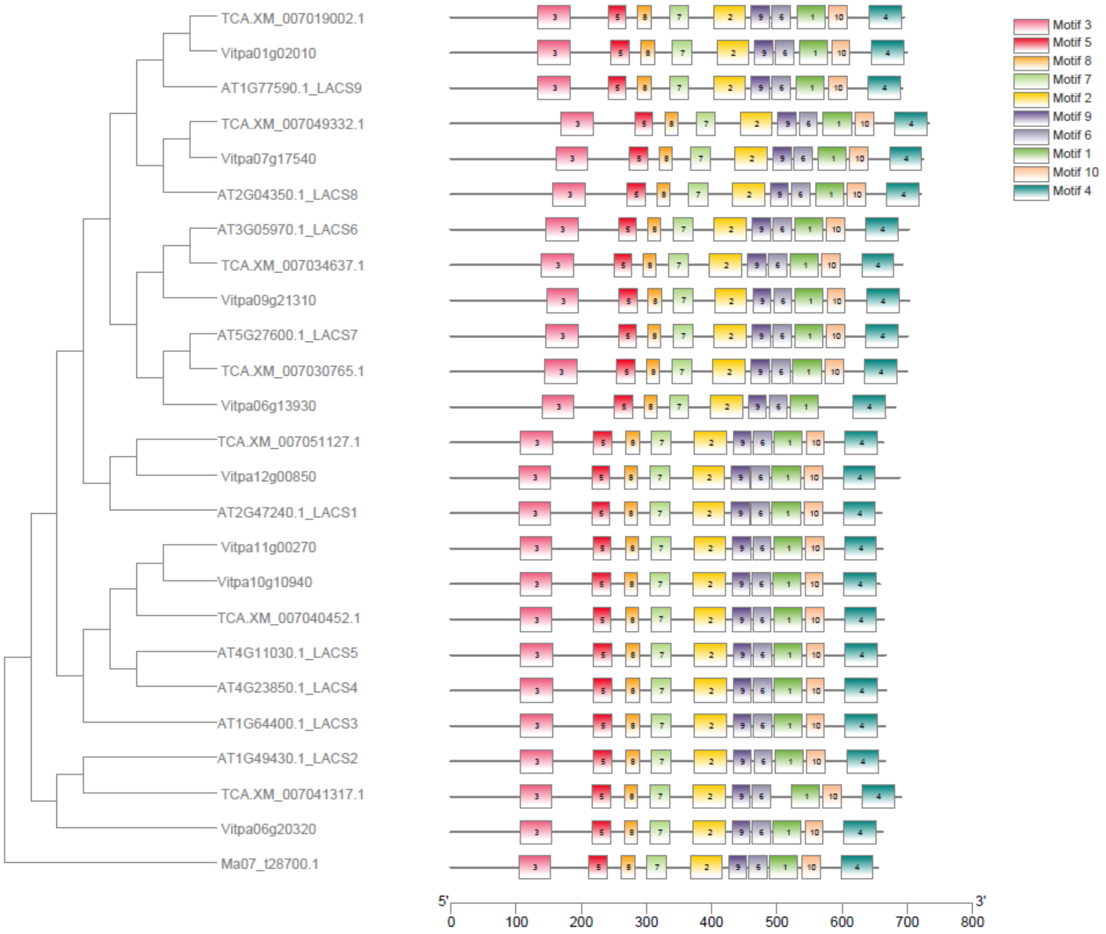

Figure S15. LACS (Long Chain Acyl-CoA Synthetase) gene tree with MEME motifs, comparing orthologs from *Arabidopsis thaliana* ("AT…"), *Theobroma cacao* ("TCA.XM…") and *Vitellaria paradoxa* ("Vitpa…"). An ortholog from *Musa acuminata* ("Ma…") of clade Monocots serves as outgroup.

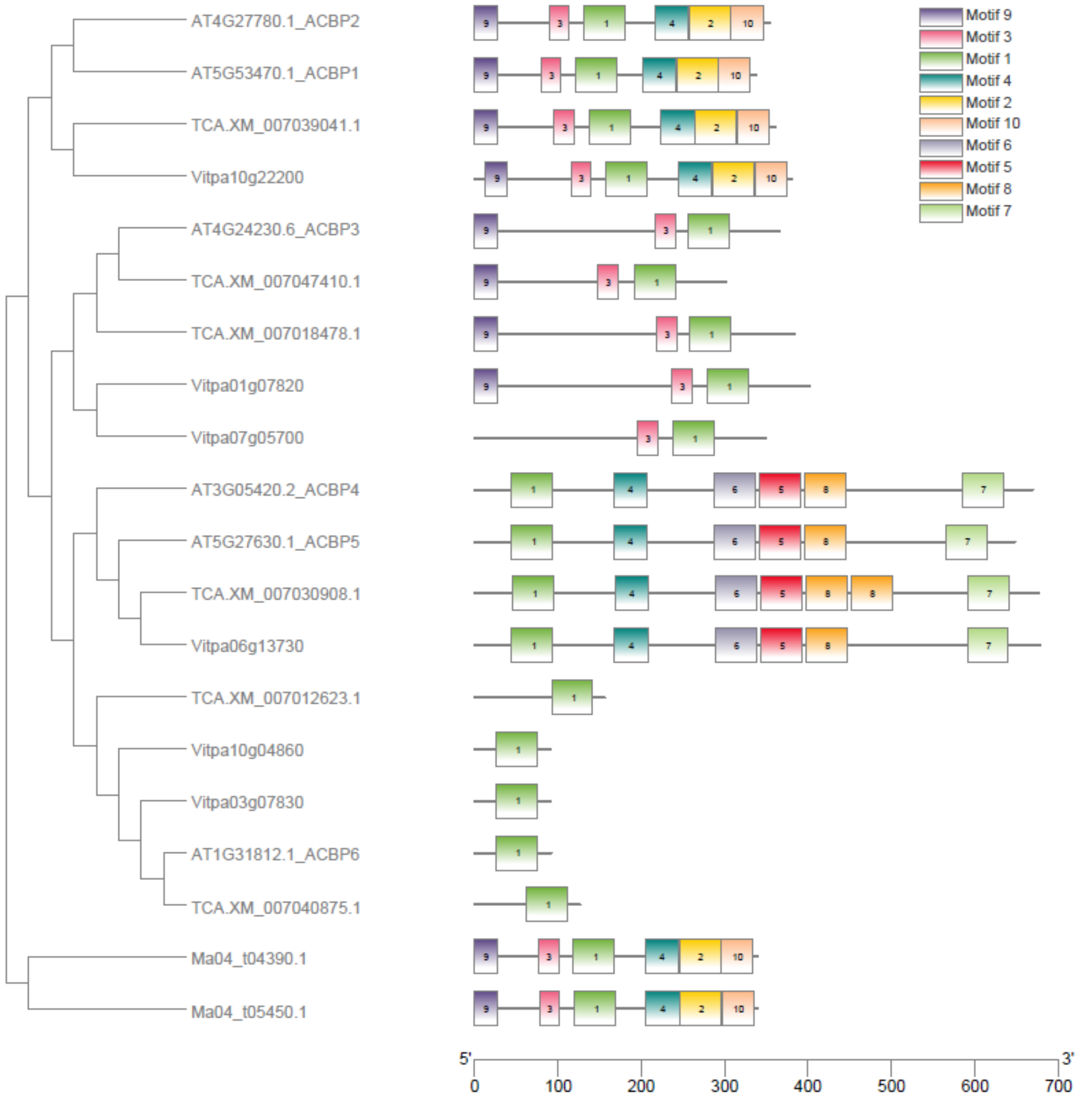

Figure S16. ACBP (Acyl-CoA-binding protein) gene tree with MEME motifs, comparing orthologs from *Arabidopsis thaliana* ("AT…"), *Theobroma cacao* ("TCA.XM…") and *Vitellaria paradoxa* ("Vitpa…"). Orthologs from *Musa acuminata* ("Ma…") of clade Monocots serve as outgroup.

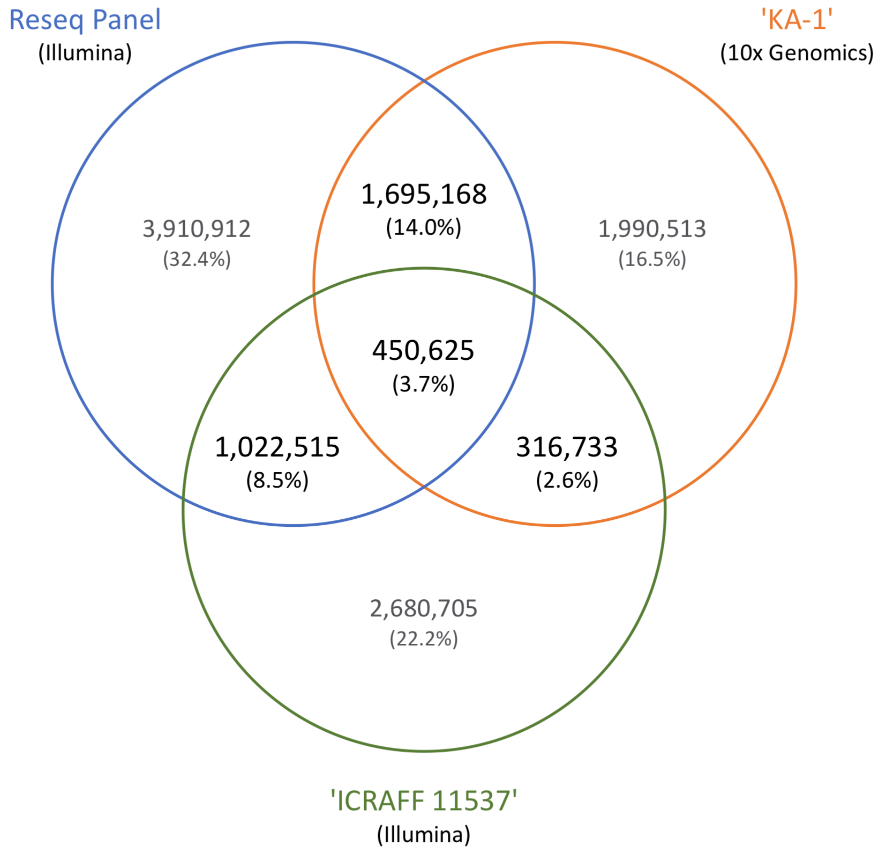

Figure S17. Venn diagram of SNPs called using the three different data sets in this study
