# Supplementary material for: Genomic Resources to Guide Improvement of the Shea Tree
Source: Front Plant Sci. 2021 Sep 9;12:720670. doi: 10.3389/fpls.2021.720670 (PMC8459026; doi:10.3389/fpls.2021.720670)
Supplement: Additional File 2: Supplementary Table 1 — Geographical provenances of the 31 shea tree accessions used in the skim resequencing panel to facilitate variant calling. [file Data_Sheet_2.PDF]

Genomic resources to guide improvement of the shea tree

Hale *et al.* 2021

Additional File 2: Supplementary Tables

Table S1. Geographical provenances of the 31 shea tree accessions used in the skim resequencing panel to facilitate variant calling

Accession ID	Country	Village/Town/Unit	Barcode	Mean Coverage
BEN1-10	Benin	Materi	AGCTACCA	1.42
BEN8-65	Benin	Tanguieta	CACAGACT	2.17
CAM03	Cameroon	Bangangte	CAACACAG	2.44
CAM04	Cameroon	Bangangte	CACAGGAA	3.35
G001	Ghana	Yendi	AGGAACAC	1.70
G007	Ghana	Yendi	AGTGACCT	1.90
G010	Ghana	Yendi	ATCGTGGT	1.63
G012	Ghana	Yendi	CAACCGTA	1.32
G015	Ghana	Yendi	CACATGGT	1.73
G021	Ghana	Yendi	CAGCATAC	3.32
G023	Ghana	Nyankpala	CATGAGCA	2.19
G026	Ghana	Nyankpala	CCGCTTAA	2.41
G032	Ghana	Nyankpala	AGGAGGTT	3.56
G048	Ghana	Walewale	ATCTCCTG	3.10
G051	Ghana	Walewale	CAACCTCT	3.05
G076	Ghana	Gulumpe	CATGGATC	2.74
G100	Ghana	Damongo	AGTGGCAA	2.83
G121	Ghana	Wa	ATCTGACC	2.68
G127	Ghana	Wa	CAACTGAC	2.94
G142	Ghana	Wa	CAGGATGT	0.69
G146	Ghana	Funsi	CATGTGTG	1.41
G155	Ghana	Walembelle	CCGTAACT	0.67
G156	Ghana	Welembelle	AGGCTGAA	1.06
G158	Ghana	Funsi	AGTTGTGC	2.58
KA04	Ghana	Bole	CCGAAGAT	2.44
SG044	Ghana	Bole	AGTGCATC	1.98
SG129	Ghana	Bole	CACCATGA	1.70
NIG01	Nigeria	Makurdi	CAGAGTGA	2.44
NIG02	Nigeria	Makurdi	CATCCAAG	3.26
NIG03	Nigeria	Buruku	CCAGTTGA	2.94
NIG05	Nigeria	Buruku	AGCTAGTG	3.24

Table S2. Filters used for SNP calling within the three datasets [10x data of reference accession 'KA01' (10x), Illumina shotgun data of 'ICRAFF 11537' (shotgun), and the skim resequencing panel consisting of 31 shea tree accessions from four countries (reseq); see Table S1]. In the filter descriptions, culling a "variant" means to completely an entire row of the vcf file (i.e. a SNP), across all accessions. In contrast, culling a "cell" means to declare as unknown a particular accession's genotypic call for a particular SNP.

Filter	Application	Description	Dataset
10X_RESCUED_MOLECULE_HIGH_DIVERSITY	Long Ranger	Culls variants supported primarily by reads that were 'rescued' with barcode-aware alignment, where the mapped molecule has a high degree of divergence from the reference. This filter reduces false-positive variant calls in complex duplicated loci that tend to have missing copies in the reference genome.	10x
10X_QUAL_FILTER	Long Ranger	A basic quality filter tuned for 10x data that culls heterozygous cells with QUAL < 15 and homozygous cells with QUAL < 50	10x
10X_ALLELE_FRACTION_FILTER	Long Ranger	A diploid-appropriate filter that culls heterozygous cells with allele fraction < 15%	10x
DP3	Custom	A basic confidence filter that culls heterozygous cells with minor allele read depth < 3	10x, shotgun
MAFraction20	Custom	A diploid-appropriate depth filter that culls variants for which the mean minor allele fraction ALT/(REF+ALT) across heterozygotes is <0.2 or >0.8	10x, shotgun
mVar_State	Custom	A basic confidence filter that culls: 1) Variants with the minor allele occuring in fewer than two individuals; and 2) Putative homozygous (HOMO_REF or HOMO_VAR) cells with read depth < 5	reseq
QUAL30	GATK QC FILTER (Empirical)	Culls variants with call quality score (QUAL) < 30. QUAL is defined as the Phred-scaled quality score for the assertion made in ALT.	10x, shotgun, reseq
QD2	GATK QC FILTER (Empirical)	Culls variants with Quality By Depth (QD) < 2.0. QD is defined as QUAL divided by unfiltered read depth from non-hom-ref samples.	10x, shotgun, reseq
FS60	GATK QC FILTER (Empirical)	Culls variants with Fisher Strand (FS) > 60.0. FS is defined as Phred-scaled probability that there is strand bias at the site, calculated by Fisher's exact test on the raw counts of reads supporting each allele on the forward and reverse strand (SB).	10x, shotgun, reseq
SOR3	GATK QC FILTER (Empirical)	Culls variants with Strand Odd Ratio (SOR) > 3.0. SOR is another way to estimate strand bias using a test similar to the symmetric odds ratio test.	10x, shotgun, reseq
MQ40	GATK QC FILTER (Empirical)	Culls variants with RMS Mapping Quality (MQ) < 40.0. MQ is defined as the square root of the average of the squares of the mapping qualities over all the reads at the site	10x, shotgun, reseq
MQRankSum-12.5	GATK QC FILTER (Empirical)	Culls variants with Mapping Quality Rank Sum Test (MQRankSum) < -12.5	10x, shotgun, reseq
ReadPosRankSum-8	GATK QC FILTER (Empirical)	Filter variants with Read Pos Rank Sum Test (ReadPosRankSum) < -8.0	10x, shotgun, reseq

Table S3. Summary of raw PacBio data obtained for *V. paradoxa* reference accession 'KA01' via 25 SMRT cells

Parameter	Value
Total data generated, Gb	162.6
Total number of reads	20,249,612
Number of reads >5kb	10,884,721
Number of reads >20kb	1,622,530
Mean read length, bp	8,142
N50 read length, bp	13,514
Maximum read length, bp	99,408

Table S4. Descriptive statistics of the various transcriptome assemblies, including the final "Integrated" assembly used for annotation

	Pure Illumina (Trinity)	Pure ONT	Hybrid (Vsearch)	Integrated
**Total size (Mbp)**	29.7	57.3	51.3	138.3
**GC content**	0.45	0.44	0.44	0.44
**No. of transcripts**	29,735	47,646	82,249	118,065
Smallest (bp)	501	500	500	500
Largest (bp)	5,116	7,267	7,267	7,267
Mean length (bp)	999	1,202	1,261	1,172
Number > 1 kbp	11,134	25,368	22,801	59,303
Length N50 (bp)	1,092	1,362	1,467	1,339
**Transcript alignment to reference (GMAP)**	99.99%	99.97%	99.97%	99.98%
**No. transcripts with ORF**	23,994	34,776	29,867	87,604
**Mean % of transcript covered by the longest ORF**	78.29	68.29	67.26	69.14
**No. of gene models**	23,961	40,483	40,684	46,868
**No. gene models with ORF**	21,869 (91.3%)	31,186 (77%)	33,595 (82.6%)	42,368 (90.4%)

Transcriptome completeness statistics (BUSCO v4.0.4 – 1,614 Embryophyta genes)

**Present**	1,191 (73.8%)	1,167 (72.3%)	1,341 (83.1%)	1,363 (84.4%)
Complete	830 (51.4%)	950 (58.9%)	1,080 (66.9%)	1,107 (68.6%)
Single-copy	667 (41.3%)	615 (38.1%)	881 (54.6%)	142 (8.8%)
Duplicated	163 (10.1%)	335 (20.8%)	199 (12.3%)	965 (59.8%)
Fragmented	361 (22.4%)	217 (13.4%)	261 (16.2%)	256 (15.9%)
**Missing**	423 (26.2%)	447 (27.7%)	273 (16.9%)	251 (15.5%)

Table S5. Structural annotation summary statistics

**Type**			**Length (bp)**	**Percentage of ‘KA-01’ reference genome (%)**
Retroelements	SINEs		150,953	0.02
	LINEs		21,229,977	3.22
	LTR	Ty1/Copia	44,890,545	6.81
		Gypsy/DIRS1	69,871,165	10.61
		Others	652,074	0.10
DNA transposons			31,182,813	4.73
Rolling-circles			2,332,964	0.35
Unclassified			210,916,928	32.02
Small RNA			616,139	0.09
Satellites			3,618,804	0.55
Simple repeats			10,062,256	1.53
Low complexity			839,424	0.13
Total			402,383,623	61.08

Table S6. K_S peak values of whole-genome duplication events detected in the Ericales

	**A*d*-α** 17.7-26.5 Mya[a]	**A*d*-β** 61.9-73.7 Mya	**A*t*-γ** 150.4-159.3 Mya
Vitis vinifera	-	-	1.05
Camellia sinensis	-	0.45	1.37
Vitellaria paradoxa	-	0.60	1.59
Rhododendron simsii	-	0.73	1.71
Actinidia chinensis	0.16	0.57	1.58

[a] Timing of WGD events following the estimates of Wu et al. (2019).

Table S7. Summary table of the 30 fatty acid biosynthesis genes from *A. thaliana*. Details can be found in the TAIR database (https://www.arabidopsis.org).

Gene	Description	TAIR Locus ID
BCCP1	Homomeric Acetyl-CoA Carboxylase BCCP subunit	AT5G16390
BCCP2	Homomeric Acetyl-CoA Carboxylase BCCP subunit	AT5G15530
CAC2	Homomeric Acetyl-CoA Carboxylase BC subunit	AT5G35360
CAC3	Homomeric Acetyl-CoA Carboxylase alpha-CT subunit	AT2G38040
KAS I	Ketoacyl-ACP synthase I	AT5G46290
KAS II	Ketoacyl-ACP synthase II	AT1G74960
KAS III	Ketoacyl-ACP synthase III	AT1G62640
FATA1	Acyl-ACP Thioesterase Fat A	AT3G25110
FATA2	Acyl-ACP Thioesterase Fat A	AT4G13050
FATB	Acyl-ACP Thioesterase Fat B	AT1G08510
FAD2	ER Oleate Desaturase	AT3G12120
FAD3	ER Linoleate Desaturase	AT2G29980
FAX1	fatty acid exporter 1	AT3G57280
FAX2	fatty acid exporter 2	AT2G38550
FAX4	fatty acid exporter 4	AT1G33265
LACS1	Long Chain Acyl-CoA Synthetase	AT2G47240
LACS2	Long Chain Acyl-CoA Synthetase	AT1G49430
LACS3	Long Chain Acyl-CoA Synthetase	AT1G64400
LACS4	Long Chain Acyl-CoA Synthetase	AT4G23850
LACS5	Long Chain Acyl-CoA Synthetase	AT4G11030
LACS6	Long Chain Acyl-CoA Synthetase	AT3G05970
LACS7	Long Chain Acyl-CoA Synthetase	AT5G27600
LACS8	Long Chain Acyl-CoA Synthetase	AT2G04350
LACS9	Long Chain Acyl-CoA Synthetase	AT1G77590
ACBP1	Acyl-CoA-binding protein	AT5G53470
ACBP2	Acyl-CoA-binding protein	AT4G27780
ACBP3	Acyl-CoA-binding protein	AT4G24230
ACBP4	Acyl-CoA-binding protein	AT3G05420
ACBP5	Acyl-CoA-binding protein	AT5G27630
ACBP6	Acyl-CoA-binding protein	AT1G31812
