# Supplementary material for: Genomic Resources to Guide Improvement of the Shea Tree
Source: Front Plant Sci. 2021 Sep 9;12:720670. doi: 10.3389/fpls.2021.720670 (PMC8459026; doi:10.3389/fpls.2021.720670)
Supplement: Additional File 3: Supplementary Text 1 — Complete FALCON command log for this study, including all specified assembly parameters. [file Data_Sheet_3.PDF]

Genomic resources to guide improvement of the shea tree

Hale *et al.* 2021

Additional File 3: Supplementary Text

Text S1 Complete FALCON command log for this study, including all specified assembly parameters

FALCON

```
[job.defaults]
njobs = 32
submit = qsub -S /bin/bash -sync y -V  \
  -q ${JOB_QUEUE}     \
  -N ${JOB_NAME}        \
  -o "${JOB_STDOUT}" \
  -e "${JOB_STDERR}" \
  -pe smp ${NPROC}    \
  "${JOB_SCRIPT}"

JOB_QUEUE = sequel-farm
MB = 4000
NPROC = 4
[General]
input_type = raw
input_fofn = subreads.fasta.fofn
pwatcher_type=blocking

# The length cutoff used for seed reads used for initial mapping
genome_size = 850000000
seed_coverage = 40
length_cutoff = 5000

# The length cutoff used for preads
length_cutoff_pr = 20000

falcon_greedy = False
falcon_sense_greedy=False

# overlapping options for Daligner
pa_HPCdaligner_option =  -v -dal128 -e0.76 -M24 -l1500 -k14 -h70 -w8 -s100
ovlp_HPCdaligner_option = -v -dal128 -M24 -k24 -h1024 -e.97 -l2500 -s100

pa_DBsplit_option = -x500 -s400
ovlp_DBsplit_option = -s400

# error correction consensus option
falcon_sense_option = --output_multi --min_idt 0.70 --min_cov 4 --max_n_read 400 --n_core 24

# overlap filtering options
overlap_filtering_setting = --max_diff 100 --max_cov 300 --min_cov 4 --n_core 24

[job.step.da]
NPROC=4
MB=32000
njobs=192
[job.step.la]
NPROC=4
MB=32000
njobs=192
[job.step.cns]
NPROC=8
MB=64000
njobs=96
[job.step.pda]
```

```
NPROC=6
MB=48000
njobs=250
[job.step.pla]
NPROC=4
MB=32000
njobs=250
[job.step.asm]
NPROC=24
MB=192000
njobs=1
```

FALCON-UNZIP

```
[General]
[Unzip]
input_fofn= subreads.fasta.fofn
input_bam_fofn=subreads.bam.fofn

[job.defaults]
pwatcher_type=blocking
max_n_open_files = 1000
njobs=100
JOB_QUEUE=bigmem
NPROC=4
MB=32768
submit = qsub -S /bin/bash -sync y -V  \
  -q ${JOB_QUEUE}     \
  -N ${JOB_NAME}       \
  -o "${JOB_STDOUT}" \
  -e "${JOB_STDERR}" \
  -pe smp ${NPROC}    \
  "${JOB_SCRIPT}"
[job.step.unzip.track_reads]
njobs=1
NPROC=24
MB=196608
[job.step.unzip.blasr_aln]
njobs=45
NPROC=24
MB=196608
[job.step.unzip.phasing]
njobs=300
NPROC=2
MB=16384
[job.step.unzip.hasm]
njobs=1
NPROC=100
MB=393216
[job.step.unzip.quiver]
njobs=100
NPROC=24
MB=199608
```

Text S2 Complete Purge Haplotigs command log for this study, including all specified parameters

```
#  https://bitbucket.org/mroachawri/purge_haplotigs/src/f6b0eea4975e534ea4e9ef29e5c9fb22b0782d5f)

$ purge_haplotigs hist -b aligned.sorted.bam -g primary_contigs.fasta

# BAM file was generated in the SMRTLink resequencing pipeline v3.2.0 by aligning all
# subreads to the FALCON-Unzip primary contigs using BLASR (Chaisson & Tesler 2012)

$ purge_haplotigs cov  -i aligned.sorted.bam.gencov  -l 11  -m 48  -h 75

$ purge_haplotigs purge -g primary_contigs.fasta -c coverage_stats.csv

# The default of 3 rounds of purging was run
```
